# Chronic airway inflammation in *Drosophila* lacking the A20-like protein Trabid

**DOI:** 10.3389/fimmu.2025.1564386

**Published:** 2025-09-05

**Authors:** Judith Bossen, Mirjam Knop, Xiao Niu, Marcus Thiedmann, Ruben Prange, Navid Tahanzadeh, Sören Franzenburg, Iris Bruchhaus, Holger Heine, Thomas Roeder

**Affiliations:** ^1^ Department Molecular Physiology, Zoology, Kiel University, Kiel, Germany; ^2^ Airway Research Center North (ARCN), Member of the German Center for Lung Research (DZL), Borstel, Germany; ^3^ Shandong Second Medical University, School of Life Sciences and Technology, Weifang, China; ^4^ Developmental Glycobiology Section, National Institute of Dental and Craniofacial Research (NIDCR), National Institutes of Health, Bethesda, MD, United States; ^5^ Institute of Clinical Molecular Biology, Kiel University, Kiel, Germany; ^6^ Host-Parasite Interaction, Bernhard-Nocht Institute for Tropical Medicine, Hamburg, Germany; ^7^ Division of Innate Immunity, Priority Research Area Chronic Lung Diseases, Research Center Borstel - Leibniz Lung Center, Borstel, Germany

**Keywords:** *Drosophila* immunity, IMD-pathway, A20, inflammation, stress resistance

## Abstract

**Background:**

Long-term disruption of epithelial immune homeostasis plays a key role in the development and persistence of chronic lung diseases. The regulation of immune pathways, especially the NF-kB pathway, is crucial for maintaining this balance. A20 (TNFAIP3) acts as an inhibitor of the NF-kB pathway and has been linked to chronic lung disease.

**Methods:**

We investigated the functional role of A20 by studying its Drosophila ortholog, Trabid. trabid-deficient flies were analyzed for immune response activation, susceptibility to various airborne stressors (dehydration, chronic cigarette smoke, and hypoxia), and their cellular adaptation to hypoxia in the tracheal system.

**Results:**

trabid-deficient Drosophila exhibited chronically activated immune responses in airway epithelial cells. These animals showed markedly increased sensitivity to dehydration, cigarette smoke, and hypoxic stress. Moreover, the plastic (adaptive) response of terminal tracheal cells to hypoxia, normally present in wild-type animals, was lost.

**Conclusion:**

Loss of A20/Trabid leads to disease-associated phenotypes resulting not only from heightened epithelial immune activity, but also from a profound reduction in the organism’s ability to withstand environmental stresses. This highlights the dual importance of immune regulation and stress resistance in epithelial health and chronic lung disease.

## 1 Introduction

The regulation of complex immune responses is of central importance for immune homeostasis and, thus, for health. In this context, factors mediating the negative regulation of immunological signaling systems play a vital role, as they effectively prevent the overactivation of immune responses. Consistently, the reduced presence of such inhibitors often leads to pathological conditions mediated by the overactivation of parts of the immune system. An example of a regulatory-acting inhibitor is the protein A20 [tumor necrosis factor α-induced protein 3 (TNFAIP3)] (1, 2). The involvement of A20 polymorphisms in several different immunological diseases has convincingly demonstrated the relevance of this NF-κB inhibitory regulator (3). These are highly diverse chronic inflammatory diseases, among which autoimmune diseases (4, 5), inflammatory bowel diseases, and various lung diseases are particularly noteworthy (6, 7).

The role of NF-κB response regulators in the context of chronic inflammatory lung diseases has attracted substantial interest. Among these diseases, asthma and COPD occupy particularly prominent roles. In both cases, non-adequate inflammatory responses occur, which are closely associated with the respective pathological conditions. Thus, decreased A20 expression correlates with increased NF-κB activity and asthma prevalence (8, 9). On the other hand, activation of A20 expression in the lung has been shown to exhibit protective effects in asthma (8, 10). The complexity of regulation is also evidenced by the fact that influenza infection has been shown to regulate microRNA 125, reducing A20 expression and increasing the inflammatory response in COPD patients (11). In addition to these direct NF-κB-mediated effects seen in various chronic inflammatory diseases of the lung, the pleiotropic effect of A20 has been demonstrated through its anti-fibrotic action by destabilizing C/EBPß in alveolar macrophages (12). Moreover, other direct and indirect targets of A20, such as IRF3, imply that it has a pleiotropic activity that also affects other processes than inflammation (13, 14). Both A20 and Trabid are deubiquitinating enzymes that affect different signaling pathways. This makes them typical examples of pleiotropic gene products. Notably, Trabid directly affects the Wnt signaling pathway by stabilizing β-catenin, thereby activating the pathway (15).

The crucial importance of A20 in the context of inflammation control has led to its establishment as a novel target of directed interventions in chronic inflammatory lung diseases (12, 16). Modeling disturbances of complex interlinked signaling systems with multiple feedback loops, such as is the case for the NF-κB/IRF3/A20 system, revealed less stability and, thus, enhanced sensitivity in response to disturbances at the level of regulatory units (17).

The fact that A20 is involved in immune homeostasis at different levels and in various cell populations through its pleiotropic action prevents a comprehensive understanding of the effect of this factor in the context of varying lung inflammatory diseases. For this reason, complementary studies using simple models with a reduced degree of complexity are helpful. We studied a corresponding *Drosophila model*. In *Drosophila*, there is an A20 ortholog called *trabid*, which has been identified as a regulator of Wnt signaling (15). However, like A20, it also functions as a negative NF-κB activation (18) regulator. *Trabid* deficiency has also been used to study the effects of an enhanced immune tonus on intestinal microbiota composition (19). We used *trabid*-deficient animals to study the effects of a chronically enhanced immune tonus on disease-related phenotypes in the *Drosophila* airways. *Drosophila* has already been highly valuable in understanding the molecular underpinnings of chronic inflammatory lung diseases (20–22). We could show the induction of a chronic immune response in *trabid*-deficient airways. Moreover, these animals showed additional phenotypes that might be directly related to chronic airway diseases. The corresponding animals showed reduced resistance to airborne stressors such as hypoxia, desiccation, or cigarette smoke. Moreover, they lost the ability to plastically respond to environmental stimuli such as hypoxia, which might also point to reduced stress resilience in these animals, a finding that was further supported by transcriptomic analyses. The current study further supports the idea that A20 is a valuable target for intervention in chronic inflammatory lung diseases such as asthma or COPD.

## 2 Results

### 2.1 Dysregulated Wnt and immune pathways in trabid-deficient trachea

**Figure 1:**
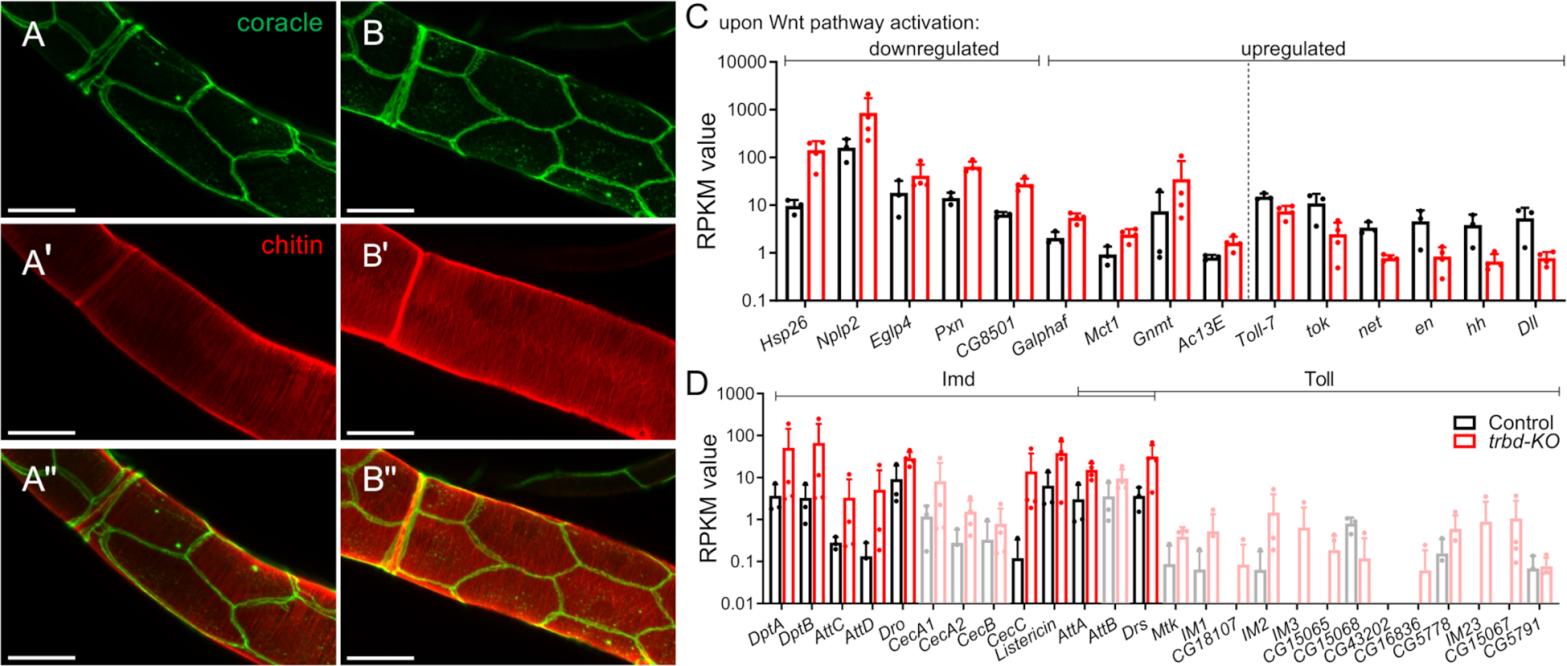
Regulation of Wnt and immune pathway target genes in the tracheal system of *trabid*-deficient animals. **(A, B)** Trachea of control animals (A, *w^1118^*) and *trabid*-deficient (*trbd-KO*) animals **(B)** stained with an antibody for the septate junction protein Coracle **(A, B)** and staining of the inner chitin layer **(Aˈ, Bˈ)**. Merge of Coracle and chitin staining is shown **(Aˈˈ, Bˈˈ)**. **(C, D)** Differentially expressed Wnt **(C)** and immune pathway **(D)** target genes. Shown are the RPKM values (mean ± SD) originating from a RNA sequencing of Control (black) and *trabid*-deficient (red) animals. The dashed line separates up- and downregulated genes. Data for genes that were not differentially expressed are shown in lighter colors.

Analysis of the airways of control (*w^1118^*) and *trabid*-deficient animals (*trbd-KO*) revealed no significant structural differences, suggesting that important developmental processes are not affected by this deletion (**Figures 1A, B**''). This is the case for the shape of the tracheae, the structure of the airway epithelial cells (**Figures 1A, B**), and the structure of the inner luminous chitin layer (**Figures 1A', B'**). We were interested in the function of Trabid in the differentiated tracheal epithelial cells and performed RNA sequencing of the dissected tissue of control and *trabid*-deficient animals. Trabid is a positive regulator of Wnt signaling (15) and a negative regulator of Imd signaling (18, 23). Initially, we wanted to confirm both these functions and looked at the target genes of both pathways (**Figures 1C, D**). Of the Wnt target genes, we selected fifteen differentially expressed genes (DEGs) that are up or downregulated upon Wnt pathway activation [**Figure 1C**; based on (24–26)]. The downregulated genes were all upregulated in *trabid*-deficient trachea (**Figure 1C**, left), whereas six of the usually ten upregulated genes were downregulated in *trabid*-deficient trachea (**Figure 1C**, right). Contrary to expectations, the remaining four genes were upregulated. Typical Wnt target genes like *wg*, *fz2*, *fz3* and *dpp* were not differentially expressed in *trabid*-deficient trachea. As Trabid is a negative regulator of Imd signaling, we expected an upregulation of numerous Imd-associated antimicrobial peptides (AMPs). We selected twenty-six AMPs that are associated with either the Imd or Toll pathways, or both (27–29). Of thirteen Imd-associated AMPs, nine were upregulated. Only two of sixteen Toll-associated AMPs were upregulated, whereby these two are also associated with IMD pathway activation (**Figure 1D**).

### 2.2 Transcriptional reprogramming and functional enrichment in trabid-deficient airways

**Figure 2:**
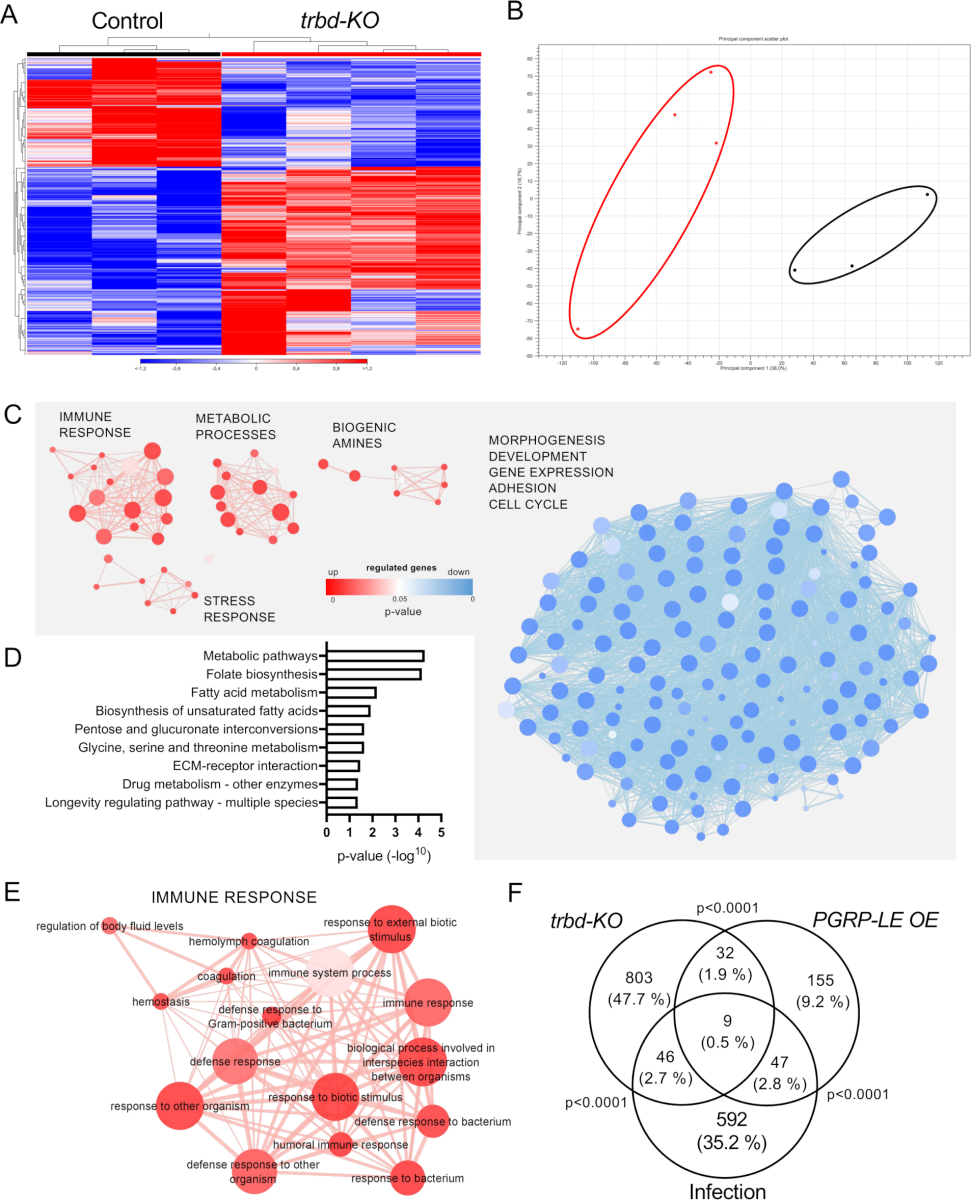
Differential gene expression profiles and enriched GO term clusters in *trabid*-deficient trachea. Trachea from control and *trabid*-deficient animals (*trbd-KO*) were used for RNAseq and transcriptomic analysis. **(A, B)** Heatmap **(A)** and PCo plot (B, circles were added manually) of the RNAseq results of the single replicates of control and *trbd-KO* trachea. **(C)** Enrichment map of biological process GO terms created from upregulated (red) and downregulated (blue) differentially expressed genes (DEGs). Node size shows the number of genes associated with the GO term, width of the connection line represents the shared genes and color intensity indicates the p-value. **(D)** KEGG pathways that are significantly associated with the DEGs. **(E)** Magnification of the cluster with GO terms that are associated with immune response created from upregulated DEGs. **(F)** Venn diagram based on DEGs from trachea of *trabid*-deficient animals, animals with tracheal infection and animals with ectopic overexpression (OE) of PGRP-LE in the trachea. Overlaps show the shared genes between the groups. Statistical significance was evaluated with Fisher’s exact test.

Further analysis of the RNA sequencing data revealed that the control and *trabid*-deficient trachea had strongly different transcriptional signatures (**Figures 2A, B**). The heat maps of the respective samples clustered depending on the origin of the samples (**Figure 2A**), and the principal component analysis showed that these samples clustered into two groups (**Figure 2B**). The subsequent gene ontology (GO) analysis based on the DEGs further showed that several GO terms were differentially modified. The significant GO terms were visualized by clustering in an enrichment map based on the shared number of genes (**Figure 2C**; **Supplementary Figures S1A, B**). GO terms based on upregulated genes are shown in red, and GO terms based on downregulated genes are shown in blue. GO terms based on upregulated genes could be separated in four clusters (**Figure 2C**). Thereof one comprised GO terms that were related to immune response, one closely related cluster comprised GO terms related to stress response, and the other two clusters were related to metabolic pathways and biogenic amines (**Figure 2C**). Downregulated genes are summarized in only one super ordinated interconnected cluster, which includes GO terms related to morphogenesis, development, gene expression, adhesion and cell cycle (**Figure 2C**, blue). Analysis of all differentially regulated genes concerning KEGG pathways revealed significance in different metabolic pathways (fatty acid, pentose, glucoronate, glycine, serine, and threonine), ECM-receptor interaction, longevity regulating pathway, and drug metabolism (**Figure 2D**). The immune response cluster shows GO terms related to defense response. Still, also to hemostasis, hemolymph coagulation, and regulation of body fluid levels (**Figure 2E**). To verify if the data resemble an actual immune response, the transcriptome results were compared to transcriptomic data from tracheal infection or ectopic overexpression (OE) of the Imd receptor PGRP-LE [**Figure 2F**, GEO database (GSE11479)], representing an Imd pathway activation (30). *Trabid-*deficient trachea showed significant overlap in the differentially expressed gene (DEG) groups to the *PGRP-LE* OE samples and infected samples. Almost 50% of the differentially expressed genes in the *trabid*-deficient trachea were altered only in this group. This shows that *trabid* deficiency leads to gene regulation in directions other than immune-related. The GO term analysis with the emergence of different biological processes also supports this.

### 2.3 Heightened sensitivity to environmental stressors in trabid-deficient flies

**Figure 3:**
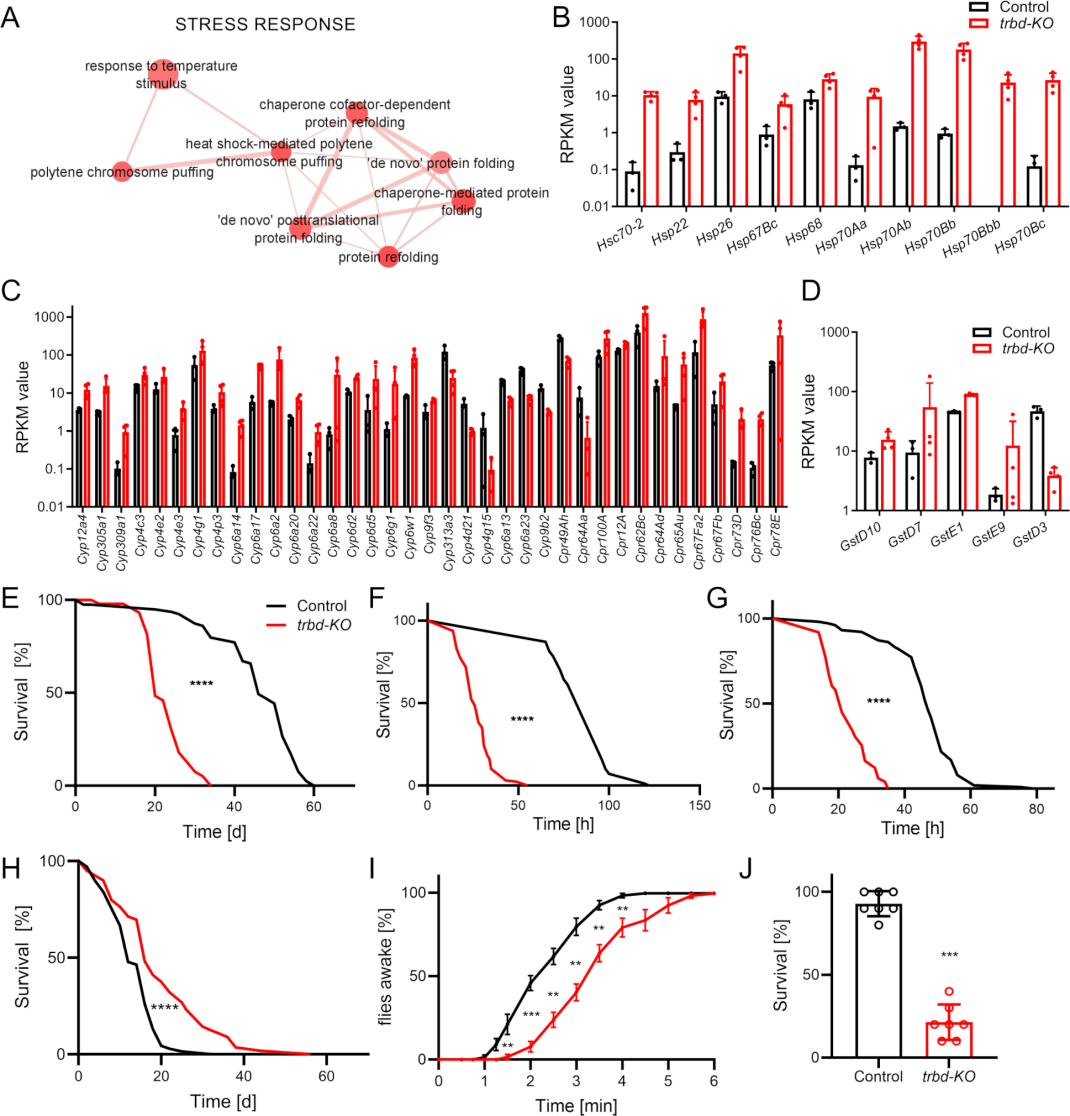
*Trabid*-deficient animals show increased sensitivity to airborne stressors. **(A)** Magnification of the Cluster with GO terms that are associated with stress response created from upregulated differentially expressed genes. **(B-D)** Differentially expressed heat shock protein genes **(B)**, cytochrome P450 related genes **(C)** and Glutathione-S-transferase genes **(D)** based on the RPKM values (mean ± SD) originating from the RNA sequencing of Control (black) and *trabid*-deficient (*trbd-KO*, red) trachea. **(E-H)** Lifespan of untreated (E, n = 54 – 79 in 5 to 7 replicates), starved (F, n = 70 – 128 in 7 to 13 replicates), desiccated (G, n = 98 – 101 in 10 replicates) and smoke exposed (H, n = 61 – 69 in 8 replicates) animals. Statistical significance in survival curves was calculated with Log rank test. **(I)** *Trabid*-deficient animals and controls were exposed to a short hypoxic shock. Recovering of the flies was recorded over time. n = 7, mean ± SEM. **(J)** Survival of *trabid*-deficient animals and controls after severe hypoxic conditions for 24 h n = 7, mean ± SD. Mann-Whitney test was used. **p<0.01, ***p<0.001, ****p<0.0001, if not otherwise specified, there was no significance.

In our further analysis, we focused on the stress response, which was enriched in *trabid*-deficient trachea (**Figure 3A**). The GO terms include protein folding related GO terms and polytene chromosome puffing. We had a look at heat shock proteins (Hsp, **Figure 3B**), cytochrome P450 proteins (Cyp; **Figure 3C**), and Glutathione S transferases (Gst; **Figure 3D**). In total, 10 Hsps were upregulated. Most Cyps among the DEGs were upregulated, while only eight were downregulated. Five Gsts were differentially expressed in the trachea of *trabid*-deficient animals, and only one was downregulated.

Subsequently, we investigated how relevant stressors acting primarily on the respiratory tract affect the lifespan of control and *trabid*-deficient animals. Under normal conditions, the life span of *trabid*-deficient animals was reduced by more approx. 40% (Control 60 days, *trbd-KO* 34 days, **Figure 3E**; Ref). The first stressor we tested was survival under starvation conditions (**Figure 3F**). *Trabid*-deficient animals showed a dramatically reduced ability to survive without access to food. Here, the median lifespan was reduced from 96 h in control to 26 h in *trabid*-deficient flies (**Figure 3F**). Another stressor, which primarily affects the airway epithelia is desiccation. Survival under very low humidity led to quick death with a median survival time of 47 h in control. In contrast, this survival time was significantly reduced to 21 h in *trabid*-deficient animals (**Figure 3G**). We subjected *trabid*-deficient *Drosophila* to a chronic smoking regimen that was characterized by confrontation with one cigarette over a period of 30 min per day. Here, *trabid*-deficient animals showed a substantially reduced lifespan under this regimen compared to matching controls. The maximum lifespan was reduced from 44 days in controls to 20 days in *trabid*-deficient animals (**Figure 3H**). Our fourth stressor known to impact airway epithelia is a lack of oxygen. In our experiments, nitrogen was introduced to replace oxygen and achieve lower oxygen content. We quantified other responses to hypoxia. Short phases of severe hypoxia subjected to adults require a specific recovery time until the animals become active again. Here, *trabid*-deficient animals recovered significantly later over three minutes if compared to the matching controls (**Figure 3I**). Prolonged hypoxia treatment in adults induces lethality that develops during the next day. The flies were subjected to severe hypoxia for 24 h, and the survival after an additional 24 h was monitored. Again, *trabid*-deficient flies show a markedly reduced survival in response to more extended hypoxia periods, with only 20% survivors compared to more than 90% in matching controls (**Figure 3J**).

### 2.4 Enhanced hypoxia sensitivity, but impaired tracheal plasticity in trabid-deficient larvae

**Figure 4:**
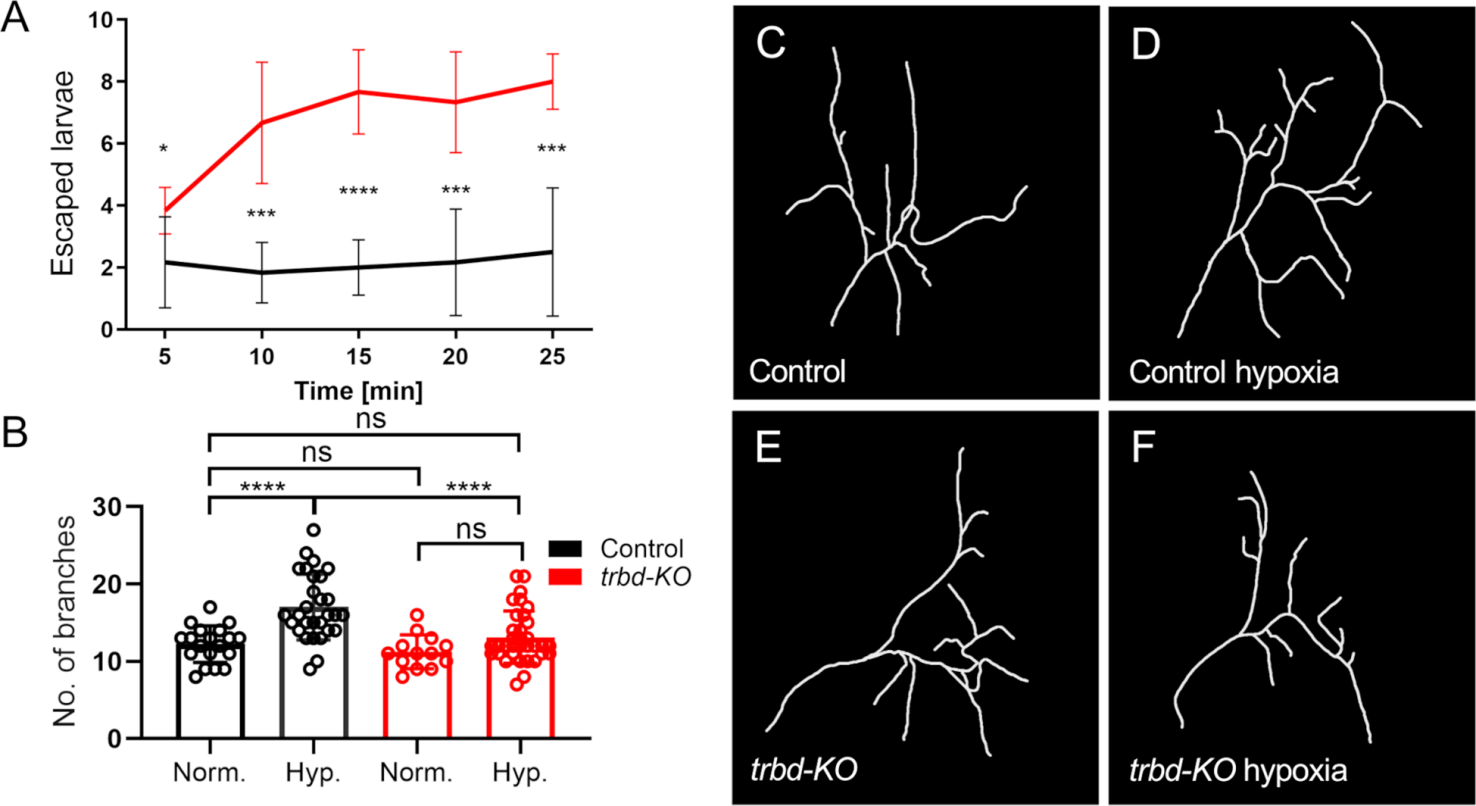
*Trabid*-deficient animals are more sensitive to hypoxia and show a defect in terminal cell branching response. **(A)** Larvae with *trabid*-deficiency (*trbd-KO*, red) and their controls (black) were exposed to hypoxic conditions of 2% O_2_. The typical behavior as a response to hypoxia was measured by determining the amount of lawn leaving larvae every five minutes. n = 6, mean ± SD. **(B)** Control and *trabid*-deficient animals were treated with 2% O_2_ for two hours. The number of terminal cell branches was quantified 24 hours later. n = 13 - 34, mean ± SD. Statistical significance was tested with Mann-Whitney test. *p<0.05, ***p<0.001, ****p<0.0001, ns, not significant. **(C–F)** Visualization of a single dorsal tracheal terminal cell (TTC) of a control animal **(C, D)** and *trabid*-deficient animal **(E, F)** under normal conditions and **(C, E)** under hypoxic conditions using the ImageJ plugin NeuronJ on TTC DIC images **(D, F)**.

We also subjected *trabid*-deficient larvae to hypoxia. Larvae show a so-called lawn-leaving response to hypoxia. The rapidity and penetrance of this response is a measure of readiness to respond to actual or perceived hypoxia. Here, at mild hypoxia of 2% O_2_, about 20% of the control animals showed this behavior, which hardly changed over time (**Figure 4A**). In contrast, *trabid*-deficient animals showed a much greater willingness to leave the medium. This effect also increased over time (**Figure 4A**). Hypoxia-induced behaviors might be associated with changes in the structure of the tracheal terminal cells that supply target tissues with oxygen. Under chronic hypoxia or increased food supply, these cells show a markedly increased formation of terminal branches responsible for providing the target tissues with oxygen (31, 32). Here, we analyzed the plastic behavior of the so-called terminal cells to hypoxia. Control and *trabid*-deficient animals did not show a difference in terminal cell structure without hypoxia (**Figures 4B, C, E**). However, the structure of the terminal cells of control animals changed under hypoxic conditions and showed an increased number of branches (**Figures 4B, D, F**). It significantly increased the number of terminal branches in control animals from approximately 12 to approximately 16. In contrast, the *trabid*-deficient animals showed no significant increase in branch number compared to the control animals under hypoxic conditions (**Figures 4B, F**). In the transcriptome data, we found no hypoxia-associated genes regulated in *trabid*-deficient trachea under normoxic conditions.

### 2.5 Biogenic amine pathways are upregulated, but melanization is impaired in trabid-deficient trachea

**Figure 5:**
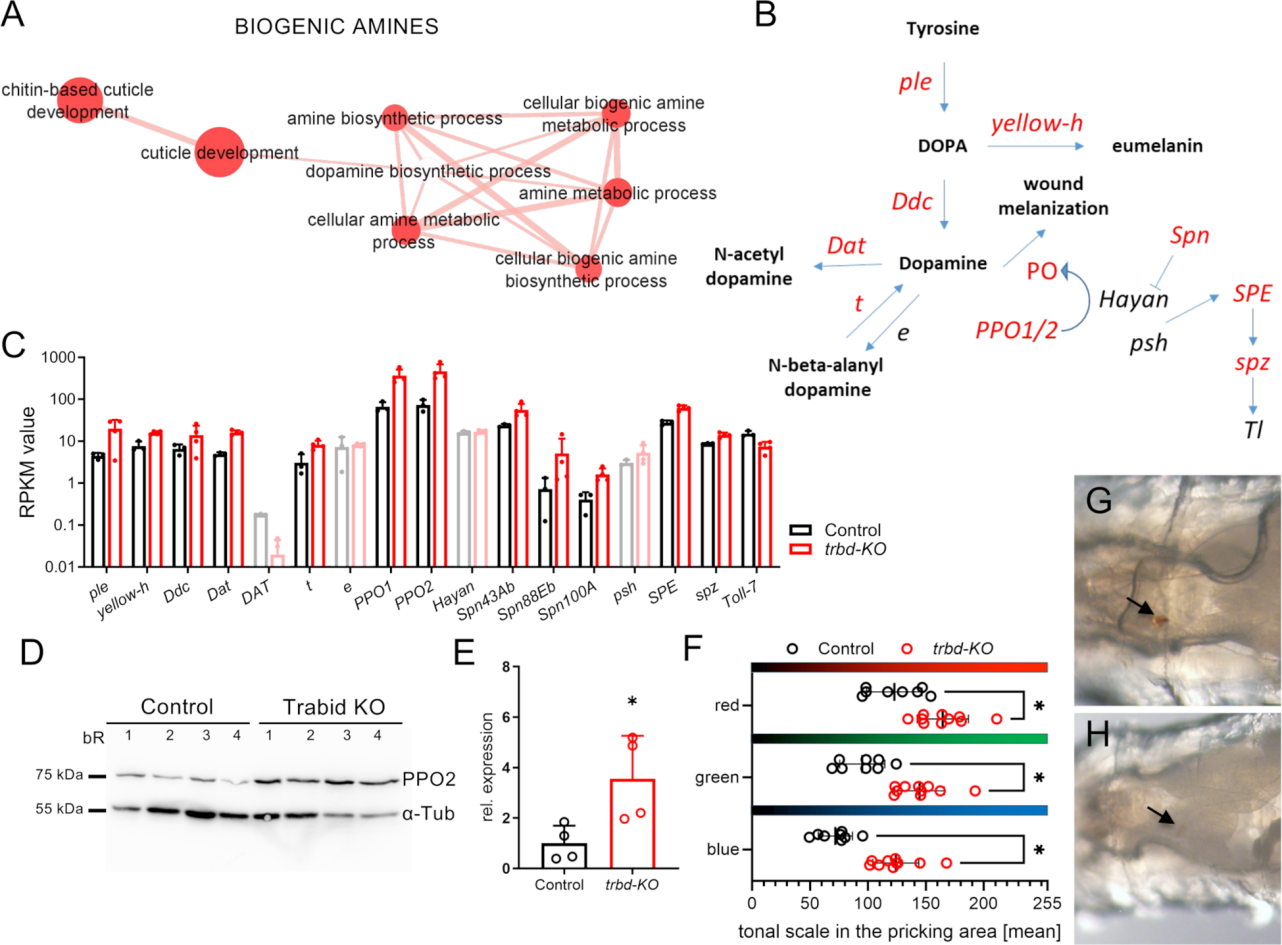
Enriched genes related to biogenic amine pathway and unchanged melanisation reaction. **(A)** Magnification of the Cluster with GO terms that are associated with biogenic amines created from upregulated differentially expressed genes (DEGs). **(B)** Schematic of dopamine biosynthetic and related processes. Genes that are differentially expressed are depicted in red. **(C)** Genes related to biogenic amine synthetic process in Control (black) and *trabid*-deficient (*trbd-KO*, red) trachea. Data for genes that were not differentially expressed are shown in lighter colors. **(D)** Western blot of Control and *trbd-KO* animals. Antibodies for prophenoloxidase 2 (PPO2) and alpha-tubulin (α-Tub) were used. bR = biological replicate. **(E)** Quantification of the PPO2 abundance relative to α -tubulin. **(F-H)** Control **(G)** and *trbd-KO* larvae **(H)** were pricked, and the melanization reaction at the pricking site (arrows) was monitored after 4 h. The color intensity was quantified in each RGB channel using the mean tonal scale with the highest pixel number in the selected area **(F)**. Mann-Whitney test, n = 8 - 10, mean ± SD.

The appearance of the GO term cluster BIOGENIC AMINES caught our attention (**Figure 5A**). It mainly contained GO terms related to the metabolic process of biogenic amines. Specifically, we focused on the steps and components of the synthesis steps from tyrosine to dopamine and related downstream processes such as melanin production and induction of Toll signaling (**Figures 5B, C**). Those genes coding for enzymes of the pathway, which are differentially expressed in the transcriptome of *trabid*-deficient trachea, are shown in red (**Figure 5B**). These include the tyrosine hydroxylase pale (ple), the dopa decarboxylase (Ddc), the dopamine acetyltransferase (Dat) and the hydrolase tan (t), which catalyze the key steps in this pathway. Melanization reactions are catalyzed by yellow-h or the phenoloxidases encoded by the *PPO1*/*PPO2* genes, all upregulated. The cleavage of prophenoloxidase to the active phenoloxidase carried out by the serine protease Hayan was not regulated. Three different serpins, that potentially inhibit the activity of Hayan were upregulated (namely *Spn43b*, *Spn88Eb*, *Spn100A*; **Figure 5C**). The Toll signaling pathway is linked to the melanization cascade via the serine proteases Hayan and Persephone (psh), which act upstream of the Spatzle-Processing Enzyme (SPE). This was upregulated in addition to the downstream Toll signaling component *spatzle* (*spz*), while the *Toll-7* receptor was downregulated (**Figures 5B, C**). We confirmed the upregulation of PPO2 at the protein level by western blot (**Figures 5D, E**). PPO2 was significantly more abundant in *trabid*-deficient animals than in the matching controls. We observed no signs of ubiquitinylation of PPO2 as an extra band with slight higher molecular mass and thus had also no differences in its deubiquitinylation through Trabid. To confirm this experimentally, we monitored the melanization reaction in control and *trabid*-deficient animals by pricking the larvae with a thin needle. After four hours, the pricking site was inspected (**Figures 5F, G**). Contrary to the expectation of a stronger melanization reaction, the pricking site was barely visible in the *trabid*-deficient animals (**Figure 5G**). The color intensity of the images was quantified in the red, green, and blue channels of the images using ImageJ (Color histogram). The mean color value with the highest number of pixels was significantly higher (brighter color scale, right) compared to the control (darker, left) in all three colors (**Figure 5H**), indicative of a reduced melanization reaction.

## 3 Discussion

Trabid, the functional ortholog of A20 in *Drosophila*, shows similar effects on NF-κB-dependent immune responses as A20 does. Trabid acts at the level of Tak1, which reduces the overall activity of pathways converging onto NF-κB. Consequently, the lack of this inhibitory input enhances immune responses. Interestingly, we observed additional changes in the transcript responses and phenotypical properties of the *trabid*-deficient trachea. Understanding these changes may shed light on why A20 deficiency impacts various diseases, which goes beyond a simple enhancement of the immune response. RNAseq analyses revealed a significant overlap of the differentially expressed genes (DEGs) between *trabid*-deficient airways and those experiencing a *PGRP-LC* overexpression. This overlap accounts for only about 15% of all DEGs from the *trabid*-deficient airways, implying that additional reactions occur without the A20 ortholog. Most importantly, we could show that the *trabid*-deficiency also increases sensitivity to stressors. We analyzed three airborne stressors, and *trabid*-deficient animals showed responses pointing in the same direction. Desiccation, cigarette smoke, and hypoxia are all far less well tolerated in *trabid*-deficient animals. Toward all these stressors, *trabid*-deficient animals show a reduced life expectancy, which is the ultimate read-out for stress sensitivity. The perceived hypoxic situation, which hardly allows the animal to tolerate hypoxic conditions, points in the same direction. Different stressors directly and indirectly influence the development of chronic inflammatory airway diseases (33). In asthma, besides psychological stress, other stressors such as cold (34), dry air (35), or cigarette smoke (36) also foster disease development. Stressors also trigger exacerbations in other chronic lung diseases, such as COPD (37). In contrast, the response to hypoxia is more complicated in asthma patients. Mild activation of a hypoxia-induced response leads to symptom improvement, whereas massive activation of the corresponding signaling pathways can be fatal (38). This is particularly relevant because intermittent hypoxia can be significant as a therapy in asthma and COPD, and A20/*trabid*-deficiency might interfere with this response (39). Our RNAseq data support this interpretation. Besides the immune response, stress-related responses are mainly induced by *trabid*-deficiency. This response might explain why these pre-stressed airways could not cope or control airways with additional stressors. Immune tone and a reduced ability to respond adequately to airborne stressors are linked to the hallmark chronic inflammatory lung diseases such as asthma and COPD (40–42). Understanding whether reduced stress resistance is a direct consequence of increased epithelial immune tone is a key area of research. However, this understanding could lead to new therapeutic opportunities, as attenuating this increased immune tone would be a useful strategy in treating these chronic inflammatory lung diseases (43, 44). The observed change in Wnt signaling in the airways also supports the link between the influence of the immune system and stress-related responses. Not only is Wnt signaling a classic target of Trabid (15), but it also plays a prominent role in developing chronic inflammatory lung diseases (45). Therefore, although the observed phenotypic changes have a cause, they are generated via different signaling pathways, which should be considered when developing possible intervention concepts.

A similar dependency as observed for the hypoxia reaction is evident between the second stressor, chronic cigarette smoking, and disease severity. For asthma (46) and COPD, we have this strong dependency on the symptom severity and the development of pathological conditions with the amount of inhaled cigarette smoke. In the case of COPD, cigarette smoke is the most critical disease inductor, and in asthma, it leads to more severe courses. In the case of COPD, in particular, genetics plays a key role in determining whether chronic cigarette smoking leads to the disease. This dependency is valid for animal models, where markedly susceptible strains contrast with others that show virtually no relevant pathogenesis (47), and for humans, where only about 20% of all smokers develop COPD (48). For COPD, A20 deficiency can have a decisive influence on the severity of symptoms, so restoration of A20 activity would be a useful therapeutic option. Identifying this as a therapeutic target is further supported by the findings of our RNAseq analyses. Here, only a few KEGG pathways were significantly enhanced, with metabolic processes and ECM-receptor interactions being the most relevant ones. This interpretation is further supported by the finding that developmental processes and those associated with adhesion are downregulated, which might indicate the inability to react suitably to structural impairments caused by exogenous noxae.

We also found substantial changes in the components of an essential arm of the immune system, the melanization cascade, in *trabid*-deficiency. This statement refers to significantly increased transcript levels of major genes encoding components of this enzyme cascade. This response is seen not only at the transcript level but also at the protein level. The expected response to this shift in the essential components of the melanization cascade would for example be the increase in melanization. However we found a decrease in melanization, suggesting the increased expression of inhibitory factors, such as the serpins mentioned above, is responsible for this paradoxical situation.

For a mechanistic understanding of the TRABID effects on the performance of the airway system, future studies should delve deeper into the mechanisms underlying the interaction between TRABID and its target signaling systems. For this, modified TRABID alleles with functional deletions of single functional domains would be promising strategy.

The current study suggests that A20/TRABID deficiency involves much more than increased immune activation. Stress-related processes that increase stress sensitivity and reduced responsiveness to environmental stimuli may be more relevant to susceptibility to chronic inflammatory lung diseases such as asthma or COPD. This interpretation is particularly pertinent in light of the potential for epigenetic changes to this system to be caused by cigarette smoke or other environmental influences (49). A20/TRABID efficiencies might change, via the reduced threshold toward these environmental stressors, the onset of epigenetic changes. These findings imply that a different treatment strategy is required, in which reducing inflammation is not necessarily the best therapeutic option; instead, directly influencing an A20 deficiency could be a more promising approach.

## 4 Material and methods

### 4.1 Drosophila strains and husbandry

*Trabid*-deficient animals were obtained from Petros Ligoxygakis (18), and the matching genetic background (*w^1118^*) were used for all experiments. *Trabid-*deficient flies were originally generated as described (15). In brief, a deletion of the *trbd* locus (CG9448) was generated by homologous recombination described in detail by Tran et al. (2008) (15). Fly husbandry was essentially performed according to earlier reports (50). Only females were used for experiments with adults. It was not differentiated in the larval stage.

### 4.2 Hypoxic exposure, desiccation, and cigarette smoke experiments

All cigarette smoking exposure experiments were carried out in a smoking chamber attached to a diaphragm pump as described before (51). Common research 3R4F cigarettes (CTRP, Kentucky University, Lexington, USA) were used for all experiments. The vials containing animals were capped with a monitoring grid to allow the cigarette smoke to diffuse into the vial. To study the effects of hypoxia, larvae were exposed to 2% hypoxia, and their lawn leaving response was quantified at the indicated times. For long-term hypoxia experiments, adults were subjected to 1.5% oxygen for 24 hours, and their survival was quantified 24 hours later. In a hypoxia-shock experiment, flies were anesthetized with N_2_ in a horizontally placed vial for 15 seconds. The time until the flies were standing up was measured.

For desiccation experiments, adults were placed into an empty vial, and their survival was monitored until all animals were dead. To examine the resistance to starvation, flies were kept in vials on 1.5% agar agar for moisture and monitored until all animals had died.

### 4.3 Antibody and tracheal stains

Larvae were dissected and fixed in 4% paraformaldehyde for 45 minutes. Immunostaining followed standard protocols described earlier (John et al., 2008, Levi et al., 2006). Coracle protein was detected with DSHB C566.9, a monoclonal mouse anti-coracle antibody (used at 1:300). Secondary antibody Alexa488-conjugated goat-anti-mouse (Jackson Immunoresearch, 1:300) was used. Tracheal chitin was stained with 505 star conjugated chitin-binding probe (NEB; 1:200). Nuclei were stained with 4’,6-Diamidino-2-Phenylindole, Dihydrochloride (DAPI) (Carl Roth GmbH HP20.1). Specimens were analyzed, and digital images were captured with a fluorescence microscope (Axio Imager.Z1 with ApoTome, Zeiss).

### 4.4 Analysis of terminal tracheal cells

Quantifying terminal branching in response to hypoxia was performed as described previously (21, 51). In brief, third instar larvae were heat-killed and placed dorsal side up on a microscopy slide. Images of the third dorsal terminal cells were taken in the DIC channel. The branches of one cell per animal were traced using the ImageJ plugin NeuronJ (52).

### 4.5 RNA isolation and RNA sequencing

For the gene expression analysis of 3rd instar larvae trachea, animals were dissected in cold PBS, isolated trachea transferred to RNA Magic (BioBudget, Krefeld, Germany) and processed essentially as described earlier (51) with slight modifications. The tissue was homogenized in a Bead Ruptor 24 (BioLab products, Bebensee, Germany) and the RNA was extracted by using the PureLink RNA Mini Kit (Thermo Fisher, Waltham, MA, USA) for phase separation with the RNA Magic reagent. An additional DNase treatment was performed following the on-column PureLink DNase treatment protocol (Thermo Fisher, Waltham, MA, USA). Total RNA input quality was evaluated on a TapeStation 4200 (Agilent, USA), and all samples showed a RIN score > 8. Samples were quantified with a fluorometric dye (Quant-IT, Thermofisher, USA) and between 187 and 500 ng per sample were used as input for the TruSeq stranded mRNA library kit (Illumina, USA) following the manufacturers manual. Resulting libraries showed a fragment size distribution of around 350 bp and were sequenced on a HiSeq 4000 (Illumina, USA) with 50 bp single-end reads. Transcriptomic analysis including gene expression values and differential expression analysis was done using CLC Genomics Workbench. The detailed protocols can be obtained from the CLC Web site (http://www.clcbio.com/products/clc-genomics-workbench). *Drosophila melanogaster* reference genome (Release 6) (53) was used for mapping. Gene Ontology enrichment analyses of the differentially expressed genes were carried out using g:Profiler (54). Data were visualized with Cytoscape enrichment map software (55).

The RNAseq data were deposited at the NCBI GEO data repository (GSE283394).

### 4.6 Western blot analysis

Female flies were homogenized in 200 µl RIPA buffer using a Bead Ruptor 24 (BioLab products, Bebensee, Germany). The protein concentration was measured with a Pierce™ BCA Protein Kit (Thermofisher, Karlruhe, Germany), and 20 µg per sample was used for protein separation by SDS-PAGE (XCell SureLock Mini-Cell Electrophoresis System, Thermofischer, Karlsruhe, Germany). The proteins were transferred from the gel to a PVDF membrane (Millipore Corporation, Bedford, MA, USA) using semi-dry transfer for one h. Unspecific binding was blocked using EveryBlot Blocking Buffer (BioRad laboratories, Feldkirchen, Germany) for one h. The membrane was incubated overnight with anti-Tubulin α antibody (AA4.3-s, DSHB) and anti-PPO2 (Won-Jae Lee, Seoul National University, Seoul, Korea) at 4 °C. After washing with TBST 3 times, the membrane was incubated with HRP-coupled anti-rabbit and anti-mouse antibodies for two hours. The bands were visualized using Clarity™ Western ECL substrate and a ChemiDocImager (BioRad laboratories, Feldkirchen, Germany).

### 4.7 Larval pricking for melanization observation

Third instar *trabid*-deficient and control animals were carefully pricked with an insect pin (Austerlitz insect pin, 0.1 mm; Pin Service, Slavkov u Brna, Czech Republic). After four hours, an RGB image of the pricking site was taken using an Olympus SZX12 stereomicroscope with an Olympus DP71 camera (Olympus Europe, Hamburg, Germany), an additional external light source [LK 1500 LCD with 2800K setting (Schott AG, Mainz, Germany)], and the cellSens entry 1.16 program. The same settings were used for all pictures: transmitted light, ISO 800, 50 ms exposure time, 24-bit RGB color (R: 0.79, G:1.00, B:1.63). The images were analyzed in ImageJ for the color intensity (mean color value with the highest number of pixels) in the selected area of the pricking site.

### 4.8 Statistical analysis

Transcriptomic analysis including gene expression values and differential expression analysis was done using CLC Genomics Workbench 8. The detailed protocols can be obtained from the CLC website (https://resources.qiagenbioinformatics.com/manuals/clcgenomicsworkbench/current/index.php?manual=Introduction_CLC_Genomics_Workbench.html). Statistical significance was controlled by FDR corrected p-value below 0.05 according to Benjamini and Hochberg (56). We used three replicates of the control and four replicates of the *trbd*-KO (**Figures 1**-**3**, **5**). For lifespan analyses, 54 – 126 animals in 5 to 13 replicates were used (**Figure 3**). Statistical significance was evaluated with Log rank test. For the hypoxia related experiments with adults a replicate number of seven was used (**Figure 3**). For the hypoxia related experiments with larvae, a replicate number of six (hypoxia sensitivity) or 13 - 34 (branching) was used (**Figure 4**). Western blot analysis was performed with four replicates per group. Melanization analysis of pricked larvae was performed with 8 – 10 animals per group (**Figure 5**; Control, *trbd*-KO). Statistical significance was evaluated by Mann-Whitney test. * p<0.05, ** p<0.01, *** p<0.001, **** p<0.0001.

### 4.9 Limitations of the study

The study was carried out using the *Drosophila* model. This has implications for the interpretation of the results. Although *Drosophila* is an excellent model, the results cannot be directly extrapolated to humans. The simplicity of the *Drosophila* system, in which there is only one innate immune system, is undoubtedly one reason for this. However, the lack of adaptive immunity is not only a disadvantage; at the same time, the unique architecture of the *Drosophila* epithelial immune system allows a clear view of the adaptive immune defense. Furthermore, it should not be forgotten that we used a *trabid* KO and thus, not only do tracheal effects contribute to the phenotypes, but possibly also other effects mediated in other organs. However, this problem was addressed by focusing on airborne stressors.

## Data Availability

The datasets presented in this study can be found in online repositories. The names of the repository/repositories and accession number(s) can be found below: https://www.ncbi.nlm.nih.gov/, GSE283394.
