# Supplementary material for: Chronic airway inflammation in Drosophila lacking the A20-like protein Trabid
Source: Front Immunol. 2025 Sep 5;16:1564386. doi: 10.3389/fimmu.2025.1564386 (PMC12446012; doi:10.3389/fimmu.2025.1564386)
Supplement: Supplementary file 1 [file DataSheet1.docx]

**Chronic airway inflammation in *Drosophila* lacking the A20-like protein trabid**

Judith Bossen^1,6,8^, Mirjam Knop^1,8^, Xiao Niu^1,2^, Marcus Thiedmann^1^, Ruben Prange^1^, Navid Tahanzadeh^1,7^, Sören Franzenburg^3^, Iris Bruchhaus^4^, Holger Heine^5,6^, Thomas Roeder^1,6*^

**Supplementary information**

**Supplementary figure**


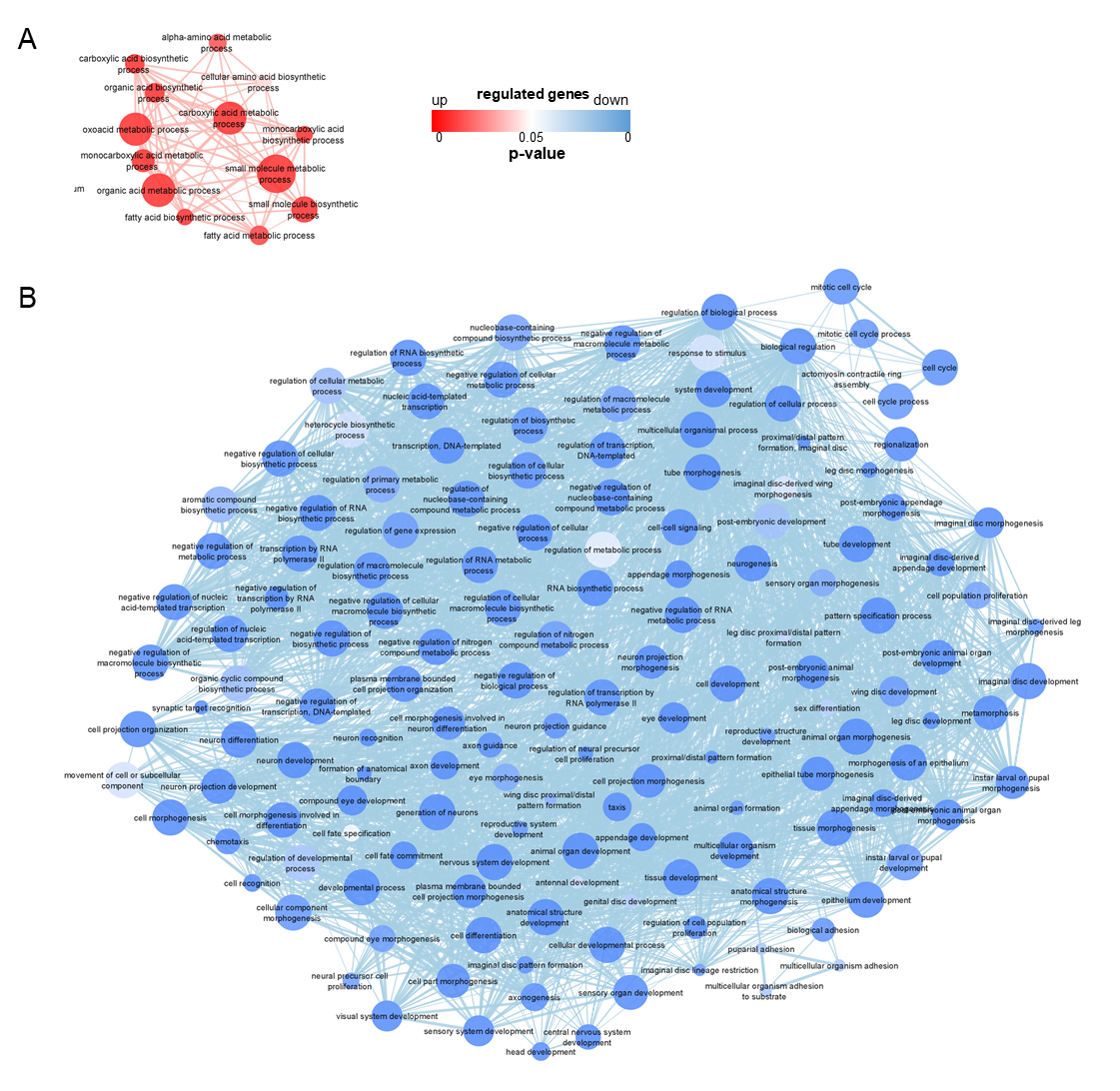


**Figure S1: Enrichment map of biological process GO terms from up- and downregulated DEGs.** GO terms related to metabolism that are generated from upregulated genes are shown in red (A). GO terms that are created from downregulated genes are shown in blue (B). Node size shows the number of genes associated with the GO term, width of the connection line represents the shared genes and colour intensity indicates the p-value.

**Supplementary tables**

Table S1: Biological Process GO terms associated with upregulated genes.

| **GO.ID** | **Description** | **p.Val** | **FDR** | **Pheno-type** | **Genes** |
| --- | --- | --- | --- | --- | --- |
| GO:0044281 | small molecule metabolic process | 1.07E-09 | 1.07E-09 | 1 | CG11659,CG6300,CYP6A8,CG9541,SODH-1,ODC1,ELO68BETA,ODC2,CG17560,ELO68ALPHA,CG30016,CG7560,CG32444,RY,CG3999,PRAT2,CG12116,CG9458,GNMT,CG8534,SMP-30,CG4302,CG17999,PLE,CG6432,ADPS,ACCOAS,ADH,CG9993,CG7860,SU(R),SRO,CG9509,GAL,SPAT,FASN2,CG15531,CG7470,AAY,CG15343,CG14893,CG3394,PDE9,TN,CG17027,PYD3,GLCAT-P,LKRSDH,MEN,DDC,ADGF-A,FAL,MPC1,PU,CG44252,CG17322,CG6746,CG3534,CG17026,GIP,TANGO14,NMDMC,CG5009 |
| GO:0016053 | organic acid biosynthetic process | 1.38E-09 | 1.38E-09 | 1 | CG11659,CG6300,ELO68BETA,ELO68ALPHA,CG7560,CG9458,GNMT,CG8534,SMP-30,CG17999,CG6432,ACCOAS,CG9993,SPAT,FASN2,CG15531,CG7470,AAY,CG3394,TN,PYD3,LKRSDH,CG44252,CG6746 |
| GO:0044283 | small molecule biosynthetic process | 3.64E-09 | 3.64E-09 | 1 | CG11659,CG6300,ELO68BETA,ELO68ALPHA,CG7560,CG12116,CG9458,GNMT,CG8534,SMP-30,CG17999,CG6432,ACCOAS,CG9993,SRO,SPAT,FASN2,CG15531,CG7470,AAY,CG15343,CG3394,PDE9,TN,PYD3,LKRSDH,ADGF-A,FAL,PU,CG44252,CG6746,TANGO14 |
| GO:0046394 | carboxylic acid biosynthetic process | 4.12E-09 | 4.12E-09 | 1 | CG11659,CG6300,ELO68BETA,ELO68ALPHA,CG7560,CG9458,GNMT,CG8534,SMP-30,CG17999,CG6432,ACCOAS,CG9993,SPAT,CG15531,CG7470,AAY,CG3394,TN,PYD3,LKRSDH,CG44252,CG6746 |
| GO:0006082 | organic acid metabolic process | 4.32E-08 | 4.32E-08 | 1 | CG11659,CG6300,CYP6A8,ODC1,ELO68BETA,ODC2,ELO68ALPHA,CG7560,RY,CG3999,CG9458,GNMT,CG8534,SMP-30,CG4302,CG17999,PLE,CG6432,ACCOAS,CG9993,CG7860,SPAT,FASN2,CG15531,CG7470,AAY,CG3394,TN,PYD3,GLCAT-P,LKRSDH,MEN,DDC,MPC1,CG44252,CG6746,GIP,NMDMC,CG5009 |
| GO:0019752 | carboxylic acid metabolic process | 7.14E-08 | 7.14E-08 | 1 | CG11659,CG6300,CYP6A8,ODC1,ELO68BETA,ODC2,ELO68ALPHA,CG7560,RY,CG3999,CG9458,GNMT,CG8534,SMP-30,CG4302,CG17999,PLE,CG6432,ACCOAS,CG9993,CG7860,SPAT,CG15531,CG7470,AAY,CG3394,TN,PYD3,GLCAT-P,LKRSDH,MEN,DDC,MPC1,CG44252,CG6746,GIP,NMDMC,CG5009 |
| GO:0043436 | oxoacid metabolic process | 9.30E-08 | 9.30E-08 | 1 | CG11659,CG6300,CYP6A8,ODC1,ELO68BETA,ODC2,ELO68ALPHA,CG7560,RY,CG3999,CG9458,GNMT,CG8534,SMP-30,CG4302,CG17999,PLE,CG6432,ACCOAS,CG9993,CG7860,SPAT,CG15531,CG7470,AAY,CG3394,TN,PYD3,GLCAT-P,LKRSDH,MEN,DDC,MPC1,CG44252,CG6746,GIP,NMDMC,CG5009 |
| GO:0050830 | defense response to Gram-positive bacterium | 3.86E-06 | 3.86E-06 | 1 | CECC,ATTD,DPTB,ATTC,SPH93,PPO2,LISTERICIN,PPO1,ATTA,DRO,EATER,SPE,SPZ |
| GO:0042335 | cuticle development | 1.59E-05 | 1.59E-05 | 1 | TWDLO,CPR76BC,CG30101,CPR73D,CPR65AU,LCP65AG2,LCP65AA,CPR67FA2,CPR64AD,TWDLP,CPR78E,LCP65AF,TWDLX,TWDLN,PLE,CPR67FB,CPR62BC,FASN2,CPR100A,T,TWDLT,CYP4G1,CCP84AB,DDC,ACP1,PU,CPR12A,EOGT |
| GO:0006959 | humoral immune response | 4.01E-05 | 4.01E-05 | 1 | CECC,DPTB,IM4,DPTA,ATTC,CAD,DRS,LECTIN-24A,PPO2,FON,NPLP2,GLT,PPO1,ATTA,DRO,HML,SPE,SPZ,CG1667 |
| GO:0032787 | monocarboxylic acid metabolic process | 4.36E-05 | 4.36E-05 | 1 | CG11659,CG6300,CYP6A8,ELO68BETA,ELO68ALPHA,CG9458,CG8534,CG4302,CG17999,CG6432,ACCOAS,CG9993,SPAT,CG15531,CG3394,TN,GLCAT-P,MEN,MPC1,CG44252,CG6746,GIP,CG5009 |
| GO:0006633 | fatty acid biosynthetic process | 5.95E-05 | 5.95E-05 | 1 | CG11659,CG6300,ELO68BETA,ELO68ALPHA,CG9458,CG8534,CG17999,CG6432,ACCOAS,CG9993,CG15531,CG3394,CG44252,CG6746 |
| GO:0072330 | monocarboxylic acid biosynthetic process | 0.000106 | 0.000106 | 1 | CG11659,CG6300,ELO68BETA,ELO68ALPHA,CG9458,CG8534,CG17999,CG6432,ACCOAS,CG9993,CG15531,CG3394,CG44252,CG6746 |
| GO:0009617 | response to bacterium | 0.000129 | 0.000129 | 1 | CECC,ATTD,YP3,DPTB,IM4,DPTA,ATTC,PIRK,DRS,SPH93,LECTIN-24A,PPO2,LISTERICIN,PPO1,ATTA,NIMC1,NIMB5,NIMB4,SR-CI,SP212,DRO,CYP6A20,EATER,NIMB2,SPE,SPZ,CG1667,SUBDUED,CG17919 |
| GO:0042026 | protein refolding | 0.000178 | 0.000178 | 1 | HSP70BBB,HSP70BC,HSP70AB,HSP70BB,HSC70-2,HSP70AA,HSP26,HSP68 |
| GO:0044106 | cellular amine metabolic process | 0.000255 | 0.000255 | 1 | W,ODC1,ODC2,RY,PPO2,PPO1,PLE,T,TN,DDC |
| GO:0009308 | amine metabolic process | 0.000255 | 0.000255 | 1 | W,ODC1,ODC2,RY,PPO2,PPO1,PLE,T,TN,DDC |
| GO:0040003 | chitin-based cuticle development | 0.000411 | 0.000411 | 1 | TWDLO,CPR76BC,CG30101,CPR73D,CPR65AU,LCP65AG2,LCP65AA,CPR67FA2,CPR64AD,TWDLP,CPR78E,LCP65AF,TWDLX,TWDLN,CPR67FB,CPR62BC,CPR100A,TWDLT,CCP84AB,ACP1,PU,CPR12A,EOGT |
| GO:0061077 | chaperone-mediated protein folding | 0.000665 | 0.000665 | 1 | HSP70BBB,HSP70BC,HSP70AB,HSP70BB,HSC70-2,HSP70AA,HSP22,HSP26,HSP67BC,HSP68,DNAJ-1 |
| GO:0009607 | response to biotic stimulus | 0.000917 | 0.000917 | 1 | CECC,ATTD,YP3,DPTB,IM4,DPTA,ATTC,PIRK,CAD,DRS,SPN88EB,SPH93,LECTIN-24A,PPO2,HF,LISTERICIN,FON,GLT,PPO1,ATTA,NIMC1,NIMB5,NIMB4,CG17738,SR-CI,SP212,DRO,CYP6A20,EATER,HML,HE,NIMB2,SPE,TEP4,SPZ,CG1667,SUBDUED,CG17919 |
| GO:0051707 | response to other organism | 0.000917 | 0.000917 | 1 | CECC,ATTD,YP3,DPTB,IM4,DPTA,ATTC,PIRK,CAD,DRS,SPN88EB,SPH93,LECTIN-24A,PPO2,HF,LISTERICIN,FON,GLT,PPO1,ATTA,NIMC1,NIMB5,NIMB4,CG17738,SR-CI,SP212,DRO,CYP6A20,EATER,HML,HE,NIMB2,SPE,TEP4,SPZ,CG1667,SUBDUED,CG17919 |
| GO:0043207 | response to external biotic stimulus | 0.000917 | 0.000917 | 1 | CECC,ATTD,YP3,DPTB,IM4,DPTA,ATTC,PIRK,CAD,DRS,SPN88EB,SPH93,LECTIN-24A,PPO2,HF,LISTERICIN,FON,GLT,PPO1,ATTA,NIMC1,NIMB5,NIMB4,CG17738,SR-CI,SP212,DRO,CYP6A20,EATER,HML,HE,NIMB2,SPE,TEP4,SPZ,CG1667,SUBDUED,CG17919 |
| GO:0006576 | cellular biogenic amine metabolic process | 0.001255 | 0.001255 | 1 | W,ODC1,ODC2,RY,PPO2,PPO1,PLE,T,DDC |
| GO:0044419 | biological process involved in interspecies interaction between organisms | 0.001418 | 0.001418 | 1 | CECC,ATTD,YP3,DPTB,IM4,DPTA,ATTC,PIRK,CAD,DRS,SPN88EB,SPH93,LECTIN-24A,PPO2,HF,LISTERICIN,FON,GLT,PPO1,ATTA,NIMC1,NIMB5,NIMB4,CG17738,SR-CI,SP212,DRO,CYP6A20,EATER,HML,HE,NIMB2,SPE,TEP4,SPZ,CG1667,SUBDUED,MVL,CG17919 |
| GO:0042742 | defense response to bacterium | 0.001997 | 0.001997 | 1 | CECC,ATTD,DPTB,DPTA,ATTC,PIRK,DRS,SPH93,LECTIN-24A,PPO2,LISTERICIN,PPO1,ATTA,NIMC1,NIMB5,NIMB4,SR-CI,DRO,CYP6A20,EATER,NIMB2,SPE,SPZ,CG1667,SUBDUED |
| GO:0035080 | heat shock-mediated polytene chromosome puffing | 0.00283 | 0.00283 | 1 | HSP70BBB,HSP70BC,HSP70AB,HSP70BB,HSP70AA |
| GO:0007599 | hemostasis | 0.00283 | 0.00283 | 1 | PPO2,FON,GLT,PPO1,HML |
| GO:0042381 | hemolymph coagulation | 0.00283 | 0.00283 | 1 | PPO2,FON,GLT,PPO1,HML |
| GO:0050817 | coagulation | 0.00283 | 0.00283 | 1 | PPO2,FON,GLT,PPO1,HML |
| GO:0042401 | cellular biogenic amine biosynthetic process | 0.004372 | 0.004372 | 1 | W,ODC1,ODC2,PLE,T,DDC |
| GO:0009309 | amine biosynthetic process | 0.004372 | 0.004372 | 1 | W,ODC1,ODC2,PLE,T,DDC |
| GO:0051085 | chaperone cofactor-dependent protein refolding | 0.004649 | 0.004649 | 1 | HSP70BBB,HSP70BC,HSP70AB,HSP70BB,HSC70-2,HSP70AA,HSP68,DNAJ-1 |
| GO:0051084 | 'de novo' posttranslational protein folding | 0.004649 | 0.004649 | 1 | HSP70BBB,HSP70BC,HSP70AB,HSP70BB,HSC70-2,HSP70AA,HSP68,DNAJ-1 |
| GO:0006631 | fatty acid metabolic process | 0.004676 | 0.004676 | 1 | CG11659,CG6300,CYP6A8,ELO68BETA,ELO68ALPHA,CG9458,CG8534,CG17999,CG6432,ACCOAS,CG9993,CG15531,CG3394,CG44252,CG6746,CG5009 |
| GO:0035079 | polytene chromosome puffing | 0.006192 | 0.006192 | 1 | HSP70BBB,HSP70BC,HSP70AB,HSP70BB,HSP70AA |
| GO:0098542 | defense response to other organism | 0.007649 | 0.007649 | 1 | CECC,ATTD,DPTB,IM4,DPTA,ATTC,PIRK,DRS,SPH93,LECTIN-24A,PPO2,HF,LISTERICIN,FON,GLT,PPO1,ATTA,NIMC1,NIMB5,NIMB4,CG17738,SR-CI,DRO,CYP6A20,EATER,HML,HE,NIMB2,SPE,TEP4,SPZ,CG1667,SUBDUED |
| GO:1901605 | alpha-amino acid metabolic process | 0.00825 | 0.00825 | 1 | ODC1,ODC2,CG7560,RY,CG3999,GNMT,PLE,CG7860,SPAT,CG7470,AAY,LKRSDH,DDC |
| GO:0006955 | immune response | 0.00966 | 0.00966 | 1 | CECC,ATTD,DPTB,IM4,DPTA,ATTC,PIRK,CAD,DRS,SPH93,LECTIN-24A,PPO2,HF,LISTERICIN,FON,NPLP2,GLT,PPO1,ATTA,NIMC1,NIMB5,NIMB4,CG17738,SR-CI,DRO,CYP6A20,EATER,HML,HE,NIMB2,SPE,TEP4,SPZ,CG1667,SUBDUED |
| GO:0009266 | response to temperature stimulus | 0.010692 | 0.010692 | 1 | HSP70BBB,HSP70BC,HSP70AB,HSP70BB,HSP70AA,FST,HSP22,HSP26,CYP6A17,HSP67BC,SMP-30,PLE,DNAJ-1,DDC,GSTE1,SUBDUED |
| GO:0050878 | regulation of body fluid levels | 0.011006 | 0.011006 | 1 | PHCL-2,PPO2,FON,GLT,PPO1,HML |
| GO:0006952 | defense response | 0.013796 | 0.013796 | 1 | CECC,ATTD,DPTB,IM4,DPTA,ATTC,PIRK,DRS,SPH93,LECTIN-24A,PPO2,HF,LISTERICIN,FON,GLT,PPO1,ATTA,NIMC1,NIMB5,NIMB4,CG17738,SR-CI,DRO,CYP6A20,EATER,HML,HE,NIMB2,SPE,TEP4,SPZ,CG1667,SUBDUED |
| GO:0006458 | 'de novo' protein folding | 0.016957 | 0.016957 | 1 | HSP70BBB,HSP70BC,HSP70AB,HSP70BB,HSC70-2,HSP70AA,HSP68,DNAJ-1 |
| GO:0008652 | cellular amino acid biosynthetic process | 0.040579 | 0.040579 | 1 | CG7560,GNMT,SPAT,CG7470,AAY,TN,PYD3,LKRSDH |
| GO:0002376 | immune system process | 0.042295 | 0.042295 | 1 | CECC,ATTD,DPTB,IM4,DPTA,ATTC,PIRK,CAD,DRS,SPH93,LECTIN-24A,PPO2,HF,LISTERICIN,FON,NPLP2,GLT,PPO1,ATTA,NIMC1,NIMB5,NIMB4,CG17738,SR-CI,DRO,CYP6A20,EATER,SRP,HML,GALPHAF,HE,NIMB2,SPE,DNAJ-1,TEP4,ADGF-A,SPZ,CG1667,SUBDUED |
| GO:0009636 | response to toxic substance | 0.042633 | 0.042633 | 1 | HSP70BC,HSP70BB,CYP6A8,ZNT35C,CG8028,CYP6G1,CYP6A2,OATP58DC,CYP6W1,CYP12A4 |
| GO:0042416 | dopamine biosynthetic process | 0.04907 | 0.04907 | 1 | PLE,T,DDC |

Table S2: Biological Process GO terms associated with downregulated genes.

| **GO.ID** | **Description** | **p.Val** | **FDR** | **Phenotype** | **Genes** |
| --- | --- | --- | --- | --- | --- |
| GO:0007399 | nervous system development | 3.64E-14 | 3.64E-14 | 1 | FT,SPI,DG,PAV,EX,TOLLO,ROBO1,TOLL-7,LAM,CHN,PBL,STAI,BABOS,POLO,CAP-D2,BRAT,SLI,MUD,BOI,DS,ESG,RL,STI,PON,CAUP,SLOW,DCP-1,L(3)NEO38,MAS,FAX,NEK2,SEMA2B,FUTSCH,OTK,AB,TEN-A,PDM3,TOK,NET,AP,TUTL,EN,TLD,HH,E(SPL)MGAMMA-HLH,NRT,DLL,NUB,E(SPL)M8-HLH,NAB,VMAT,NWK,DYSC,TEY,E(SPL)MDELTA-HLH,PDM2,WNT4,ROBO3,HIG,SCA,DSCAM4,DAN,CG15765,WRAPPER,GL,LOV,BTD,OPTIX,GAD1,ATO,SIDPN,DAC,CAS,TOY,DANR,NERFIN-1 |
| GO:0048731 | system development | 1.77E-13 | 1.77E-13 | 1 | BARK,FT,SPI,DG,PAV,YTR,EX,TOLLO,ROBO1,TOLL-7,LAM,CHN,PBL,STAI,BABOS,POLO,CAP-D2,RHOGAP54D,JUMU,BRAT,SLI,MUD,BOI,DS,ESG,TINA-1,RL,STI,PON,CAUP,SLOW,CKS30A,DCP-1,TYN,L(3)NEO38,MAS,FAX,NEK2,SEMA2B,FUTSCH,OTK,TSH,AB,TEN-A,PDM3,TOK,NET,AP,IA-2,TIO,TOM,TUTL,EN,TLD,HH,SOX15,E(SPL)MGAMMA-HLH,NRT,DLL,NUB,AL,DVE,E(SPL)M8-HLH,NAB,VMAT,NWK,DYSC,RN,TEY,DOC1,E(SPL)MDELTA-HLH,VG,PDM2,E(SPL)M4-BFM,WNT4,ROBO3,HIG,DOC2,SCA,DSCAM4,SCR,DAN,CG15765,WRAPPER,SP1,GL,LOV,BTD,OPTIX,GAD1,TFAP-2,ATO,SIDPN,DAC,CAS,TOY,DANR,NERFIN-1 |
| GO:0048699 | generation of neurons | 1.59E-12 | 1.59E-12 | 1 | FT,SPI,DG,PAV,EX,TOLLO,ROBO1,TOLL-7,CHN,PBL,STAI,BABOS,POLO,BRAT,SLI,MUD,BOI,DS,ESG,RL,PON,CAUP,SLOW,DCP-1,MAS,FAX,NEK2,SEMA2B,FUTSCH,OTK,AB,TEN-A,PDM3,TOK,NET,AP,TUTL,EN,TLD,HH,E(SPL)MGAMMA-HLH,NRT,NUB,E(SPL)M8-HLH,NAB,VMAT,DYSC,TEY,E(SPL)MDELTA-HLH,PDM2,WNT4,ROBO3,HIG,DSCAM4,WRAPPER,GL,BTD,OPTIX,ATO,SIDPN,DAC,CAS,NERFIN-1 |
| GO:0022008 | neurogenesis | 1.59E-11 | 1.59E-11 | 1 | FT,SPI,DG,PAV,EX,TOLLO,ROBO1,TOLL-7,CHN,PBL,STAI,BABOS,POLO,BRAT,SLI,MUD,BOI,DS,ESG,RL,PON,CAUP,SLOW,DCP-1,MAS,FAX,NEK2,SEMA2B,FUTSCH,OTK,AB,TEN-A,PDM3,TOK,NET,AP,TUTL,EN,TLD,HH,E(SPL)MGAMMA-HLH,NRT,NUB,E(SPL)M8-HLH,NAB,VMAT,DYSC,TEY,E(SPL)MDELTA-HLH,PDM2,WNT4,ROBO3,HIG,DSCAM4,WRAPPER,GL,BTD,OPTIX,ATO,SIDPN,DAC,CAS,NERFIN-1 |
| GO:0032501 | multicellular organismal process | 3.00E-11 | 3.00E-11 | 1 | BARK,FT,SPI,SSRP,DG,PAV,YTR,EX,TOLLO,ROBO1,TOLL-7,LAM,DCP2,CHN,PBL,KLP61F,STAI,INCENP,BABOS,POLO,MCM6,CAP-D2,FZY,RHOGAP54D,JUMU,KLP67A,BRAT,EIP63E,SLI,MUD,BOI,DS,ESG,TINA-1,RL,TEFU,CG17211,STI,PON,CAUP,SLOW,CKS30A,DCP-1,TYN,PIGS,L(3)NEO38,MAS,FAX,NEK2,SEMA2B,FUTSCH,OTK,TSH,AB,CPR49AH,TEN-A,SKIP,TET,PDM3,TOK,NET,AP,CG18067,IA-2,CG15497,TIO,TOM,TUTL,EN,TLD,HH,SOX15,SDR,MIPLE1,E(SPL)MGAMMA-HLH,NRT,CG2650,OBP57D,FNE,DLL,NUB,AL,DPR16,DVE,RPK,E(SPL)M8-HLH,NAB,VMAT,NWK,DYSC,RN,NA,TEY,DOC1,DOPECR,E(SPL)MDELTA-HLH,VG,CCKLR-17D3,COROLLA,LCP9,PDM2,GABA-B-R3,E(SPL)M4-BFM,SMOG,WNT4,NMDAR1,CPR64AA,ROBO3,HIG,WRY,DOC2,GLUCLALPHA,SCA,DSCAM4,DOP1R2,SCR,CG3078,DAN,CG15765,SGS7,NACHRBETA2,NPF,NACHRALPHA1,WRAPPER,SP1,GL,LOV,BTD,OPTIX,SNPF,GAD1,TFAP-2,ATO,SIDPN,NACHRBETA1,DAC,OBP44A,NACHRALPHA5,CAS,NACHRALPHA6,TUT,TOY,DANR,EIG71EE,SGS3,SGS5,NERFIN-1,HUG |
| GO:0030182 | neuron differentiation | 3.18E-11 | 3.18E-11 | 1 | FT,SPI,DG,PAV,EX,TOLLO,ROBO1,TOLL-7,CHN,PBL,STAI,BABOS,BRAT,SLI,MUD,BOI,DS,RL,CAUP,SLOW,DCP-1,MAS,FAX,NEK2,SEMA2B,FUTSCH,OTK,AB,TEN-A,PDM3,TOK,NET,AP,TUTL,EN,TLD,HH,NRT,NUB,E(SPL)M8-HLH,NAB,VMAT,DYSC,TEY,E(SPL)MDELTA-HLH,WNT4,ROBO3,HIG,DSCAM4,WRAPPER,GL,BTD,OPTIX,ATO,DAC,CAS,NERFIN-1 |
| GO:0048513 | animal organ development | 3.33E-11 | 3.33E-11 | 1 | FT,SPI,DG,YTR,EX,ROBO1,LAM,CHN,PBL,POLO,CAP-D2,RHOGAP54D,JUMU,BRAT,SLI,MUD,BOI,DS,ESG,TINA-1,RL,STI,PON,CAUP,SLOW,CKS30A,TYN,L(3)NEO38,MAS,NEK2,SEMA2B,FUTSCH,OTK,TSH,AB,TEN-A,TOK,NET,AP,TIO,TOM,TUTL,EN,TLD,HH,SOX15,E(SPL)MGAMMA-HLH,DLL,AL,DVE,E(SPL)M8-HLH,NAB,DYSC,RN,DOC1,E(SPL)MDELTA-HLH,VG,E(SPL)M4-BFM,WNT4,ROBO3,DOC2,SCA,SCR,DAN,SP1,GL,BTD,OPTIX,TFAP-2,ATO,DAC,CAS,TOY,DANR |
| GO:0048869 | cellular developmental process | 9.12E-10 | 9.12E-10 | 1 | FT,SPI,SSRP,DG,PAV,YTR,EX,TOLLO,ROBO1,TOLL-7,LAM,DCP2,CHN,PBL,STAI,BABOS,POLO,MCM6,SMC2,BRAT,SLI,MUD,BOI,DS,ESG,RL,TEFU,PON,CAUP,SLOW,DCP-1,TYN,PIGS,MAS,FAX,NEK2,SEMA2B,FUTSCH,OTK,TSH,AB,TEN-A,TET,PDM3,TOK,NET,AP,TIO,TOM,TUTL,EN,TLD,HH,SOX15,E(SPL)MGAMMA-HLH,NRT,NUB,E(SPL)M8-HLH,NAB,VMAT,DYSC,RN,TEY,DOC1,E(SPL)MDELTA-HLH,PDM2,E(SPL)M4-BFM,SMOG,BRD,WNT4,ROBO3,HIG,DOC2,DSCAM4,FD96CA,WRAPPER,GL,LOV,BTD,OPTIX,FD96CB,ATO,SIDPN,DAC,CAS,TUT,NERFIN-1 |
| GO:0030154 | cell differentiation | 1.74E-09 | 1.74E-09 | 1 | FT,SPI,SSRP,DG,PAV,YTR,EX,TOLLO,ROBO1,TOLL-7,DCP2,CHN,PBL,STAI,BABOS,POLO,MCM6,SMC2,BRAT,SLI,MUD,BOI,DS,ESG,RL,TEFU,PON,CAUP,SLOW,DCP-1,TYN,PIGS,MAS,FAX,NEK2,SEMA2B,FUTSCH,OTK,TSH,AB,TEN-A,TET,PDM3,TOK,NET,AP,TIO,TOM,TUTL,EN,TLD,HH,SOX15,E(SPL)MGAMMA-HLH,NRT,NUB,E(SPL)M8-HLH,NAB,VMAT,DYSC,RN,TEY,DOC1,E(SPL)MDELTA-HLH,PDM2,E(SPL)M4-BFM,SMOG,BRD,WNT4,ROBO3,HIG,DOC2,DSCAM4,FD96CA,WRAPPER,GL,LOV,BTD,OPTIX,FD96CB,ATO,SIDPN,DAC,CAS,TUT,NERFIN-1 |
| GO:0035295 | tube development | 1.02E-08 | 1.02E-08 | 1 | FT,SPI,DG,EX,ROBO1,LAM,PBL,POLO,RHOGAP54D,JUMU,SLI,BOI,DS,ESG,RL,CG17211,PON,CAUP,CKS30A,TYN,OTK,TSH,TOK,NET,AP,IA-2,TIO,EN,TLD,HH,SOX15,E(SPL)MGAMMA-HLH,DLL,AL,DVE,NAB,RN,DOC1,VG,DOC2,SCA,SCR,DAN,SP1,BTD,TFAP-2,ATO,DAC,TOY,DANR |
| GO:0007275 | multicellular organism development | 1.20E-08 | 1.20E-08 | 1 | BARK,FT,SPI,DG,PAV,YTR,EX,TOLLO,ROBO1,TOLL-7,LAM,DCP2,CHN,PBL,STAI,BABOS,POLO,CAP-D2,RHOGAP54D,JUMU,BRAT,EIP63E,SLI,MUD,BOI,DS,ESG,TINA-1,RL,CG17211,STI,PON,CAUP,SLOW,CKS30A,DCP-1,TYN,L(3)NEO38,MAS,FAX,NEK2,SEMA2B,FUTSCH,OTK,TSH,AB,CPR49AH,TEN-A,PDM3,TOK,NET,AP,IA-2,TIO,TOM,TUTL,EN,TLD,HH,SOX15,MIPLE1,E(SPL)MGAMMA-HLH,NRT,DLL,NUB,AL,DVE,E(SPL)M8-HLH,NAB,VMAT,NWK,DYSC,RN,TEY,DOC1,E(SPL)MDELTA-HLH,VG,LCP9,PDM2,E(SPL)M4-BFM,SMOG,WNT4,CPR64AA,ROBO3,HIG,DOC2,SCA,DSCAM4,SCR,DAN,CG15765,WRAPPER,SP1,GL,LOV,BTD,OPTIX,SNPF,GAD1,TFAP-2,ATO,SIDPN,DAC,CAS,TOY,DANR,NERFIN-1 |
| GO:0032502 | developmental process | 6.05E-08 | 6.05E-08 | 1 | BARK,FT,SPI,SSRP,DG,PAV,YTR,EX,TOLLO,ROBO1,TOLL-7,LAM,DCP2,CHN,PBL,STAI,BABOS,POLO,MCM6,CAP-D2,SMC2,FZY,RHOGAP54D,JUMU,BRAT,EIP63E,SLI,MUD,BOI,DS,ESG,TINA-1,RL,TEFU,CG17211,STI,PON,CAUP,SLOW,CKS30A,DCP-1,TYN,PIGS,L(3)NEO38,MAS,FAX,NEK2,SEMA2B,FUTSCH,OTK,TSH,AB,CPR49AH,TEN-A,TET,PDM3,TOK,NET,AP,IA-2,TIO,TOM,TUTL,EN,TLD,HH,SOX15,SDR,MIPLE1,E(SPL)MGAMMA-HLH,NRT,DLL,NUB,AL,DVE,E(SPL)M8-HLH,NAB,VMAT,NWK,DYSC,RN,TEY,DOC1,E(SPL)MDELTA-HLH,VG,LCP9,PDM2,E(SPL)M4-BFM,SMOG,BRD,WNT4,CPR64AA,ROBO3,HIG,DOC2,SCA,DSCAM4,DOP1R2,SCR,DAN,FD96CA,CG15765,WRAPPER,SP1,GL,LOV,BTD,OPTIX,SNPF,GAD1,TFAP-2,FD96CB,ATO,SIDPN,DAC,CAS,TUT,TOY,DANR,NERFIN-1 |
| GO:0048856 | anatomical structure development | 6.64E-08 | 6.64E-08 | 1 | BARK,FT,SPI,SSRP,DG,PAV,YTR,EX,TOLLO,ROBO1,TOLL-7,LAM,DCP2,CHN,PBL,STAI,BABOS,POLO,MCM6,CAP-D2,RHOGAP54D,JUMU,BRAT,EIP63E,SLI,MUD,BOI,DS,ESG,TINA-1,RL,TEFU,CG17211,STI,PON,CAUP,SLOW,CKS30A,DCP-1,TYN,PIGS,L(3)NEO38,MAS,FAX,NEK2,SEMA2B,FUTSCH,OTK,TSH,AB,CPR49AH,TEN-A,TET,PDM3,TOK,NET,AP,IA-2,TIO,TOM,TUTL,EN,TLD,HH,SOX15,MIPLE1,E(SPL)MGAMMA-HLH,NRT,DLL,NUB,AL,DVE,E(SPL)M8-HLH,NAB,VMAT,NWK,DYSC,RN,TEY,DOC1,E(SPL)MDELTA-HLH,VG,LCP9,PDM2,E(SPL)M4-BFM,SMOG,WNT4,CPR64AA,ROBO3,HIG,DOC2,SCA,DSCAM4,SCR,DAN,FD96CA,CG15765,WRAPPER,SP1,GL,LOV,BTD,OPTIX,SNPF,GAD1,TFAP-2,FD96CB,ATO,SIDPN,DAC,CAS,TUT,TOY,DANR,NERFIN-1 |
| GO:0009888 | tissue development | 6.91E-08 | 6.91E-08 | 1 | FT,SPI,SSRP,DG,EX,TOLLO,ROBO1,LAM,PBL,STAI,POLO,MCM6,RHOGAP54D,JUMU,EIP63E,SLI,BOI,DS,ESG,RL,CG17211,PON,CAUP,CKS30A,TYN,PIGS,SEMA2B,OTK,TSH,AB,TOK,NET,AP,TIO,EN,TLD,HH,SOX15,MIPLE1,E(SPL)MGAMMA-HLH,DLL,AL,DVE,E(SPL)M8-HLH,NAB,RN,DOC1,VG,PDM2,SMOG,WNT4,DOC2,SCA,DAN,SP1,BTD,TFAP-2,ATO,DAC,TOY,DANR |
| GO:0009653 | anatomical structure morphogenesis | 1.18E-07 | 1.18E-07 | 1 | FT,SPI,DG,PAV,EX,TOLLO,ROBO1,TOLL-7,LAM,CHN,PBL,BABOS,MCM6,RHOGAP54D,JUMU,EIP63E,SLI,MUD,BOI,DS,ESG,RL,CG17211,STI,CAUP,CKS30A,TYN,PIGS,FAX,NEK2,SEMA2B,FUTSCH,OTK,TSH,AB,TEN-A,PDM3,TOK,NET,AP,TIO,TOM,TUTL,EN,TLD,HH,SOX15,MIPLE1,NRT,DLL,NUB,AL,DVE,E(SPL)M8-HLH,DYSC,RN,E(SPL)MDELTA-HLH,VG,SMOG,WNT4,ROBO3,SCA,DSCAM4,SCR,DAN,FD96CA,SP1,GL,BTD,OPTIX,TFAP-2,FD96CB,ATO,DAC,TOY,DANR,NERFIN-1 |
| GO:0060429 | epithelium development | 1.82E-07 | 1.82E-07 | 1 | FT,SPI,SSRP,DG,EX,TOLLO,ROBO1,LAM,PBL,STAI,POLO,MCM6,RHOGAP54D,JUMU,EIP63E,SLI,BOI,DS,ESG,RL,CG17211,PON,CAUP,CKS30A,TYN,PIGS,OTK,TSH,AB,TOK,NET,AP,TIO,EN,TLD,HH,SOX15,E(SPL)MGAMMA-HLH,DLL,AL,DVE,NAB,RN,DOC1,VG,SMOG,WNT4,DOC2,SCA,DAN,SP1,BTD,TFAP-2,ATO,DAC,TOY,DANR |
| GO:0035107 | appendage morphogenesis | 4.43E-07 | 4.43E-07 | 1 | FT,SPI,DG,RHOGAP54D,JUMU,DS,RL,CAUP,TYN,TSH,TOK,NET,AP,EN,TLD,HH,DLL,NUB,AL,DVE,RN,VG,SCA,DAN,SP1,BTD,TFAP-2,ATO,DAC,DANR |
| GO:0050789 | regulation of biological process | 7.12E-07 | 7.12E-07 | 1 | FT,SPI,SSRP,DG,CG9135,PAV,YETI,EX,TOLLO,ROBO1,CG40191,TOLL-7,LAM,DCP2,CHN,PBL,KLP61F,STAI,INCENP,BABOS,POLO,CG1965,SMC2,CG9231,FZY,CG9125,RHOGAP54D,JUMU,BRAT,MARS,EIP63E,SLI,BOI,DS,ESG,RL,TEFU,STI,ROD,MSH6,RHOGEF4,CAUP,CG12299,CKS30A,CADPS,DCP-1,TYN,PIGS,L(3)NEO38,MAS,CG43366,NEK2,SEMA2B,FUTSCH,OTK,CG5466,TSH,AB,TEN-A,TET,PDM3,TOK,NET,AP,TRBD,CG13928,IA-2,CG6654,MRE11,RAB27,TIO,TOM,TUTL,EN,TLD,HH,SOX15,SDR,E(SPL)MGAMMA-HLH,CG2650,FNE,DLL,NUB,AL,DVE,E(SPL)M8-HLH,NAB,VMAT,NWK,DYSC,RN,NA,TEY,DOC1,CG11438,DOPECR,E(SPL)MDELTA-HLH,VG,CCKLR-17D3,CG43347,PDM2,GABA-B-R3,E(SPL)M4-BFM,SMOG,BRD,WNT4,NMDAR1,WRY,CG10151,DOC2,GLUCLALPHA,DOP1R2,SCR,CG3078,DAN,FD96CA,OCHO,NACHRBETA2,CNGL,NPF,NACHRALPHA1,SP1,GYC89DB,GL,UNC-4,LOV,BTD,OPTIX,SNPF,SCRT,TFAP-2,FD96CB,ATO,SIDPN,NACHRBETA1,DAC,NACHRALPHA5,CAS,NACHRALPHA6,TUT,TOY,DANR,VGLUT,NERFIN-1,HUG,ADA1-1 |
| GO:0048736 | appendage development | 7.42E-07 | 7.42E-07 | 1 | FT,SPI,DG,RHOGAP54D,JUMU,DS,RL,CAUP,TYN,TSH,TOK,NET,AP,EN,TLD,HH,DLL,NUB,AL,DVE,RN,VG,SCA,DAN,SP1,BTD,TFAP-2,ATO,DAC,DANR |
| GO:0009887 | animal organ morphogenesis | 1.2356E-06 | 1.2356E-06 | 1 | FT,SPI,DG,EX,ROBO1,LAM,CHN,RHOGAP54D,JUMU,SLI,BOI,DS,ESG,RL,CAUP,CKS30A,TYN,NEK2,OTK,TSH,TOK,NET,AP,TOM,TUTL,EN,TLD,HH,DLL,AL,DVE,E(SPL)M8-HLH,DYSC,RN,E(SPL)MDELTA-HLH,VG,WNT4,SCA,SCR,SP1,GL,BTD,OPTIX,TFAP-2,ATO,DAC,TOY |
| GO:0050794 | regulation of cellular process | 1.5473E-06 | 1.5473E-06 | 1 | FT,SPI,SSRP,DG,PAV,YETI,EX,TOLLO,ROBO1,CG40191,TOLL-7,LAM,DCP2,CHN,PBL,KLP61F,STAI,INCENP,BABOS,POLO,CG1965,SMC2,CG9231,FZY,RHOGAP54D,JUMU,BRAT,MARS,EIP63E,SLI,BOI,DS,ESG,RL,TEFU,STI,ROD,MSH6,RHOGEF4,CAUP,CG12299,CKS30A,CADPS,DCP-1,PIGS,L(3)NEO38,MAS,CG43366,NEK2,SEMA2B,FUTSCH,OTK,CG5466,TSH,AB,TEN-A,TET,PDM3,TOK,NET,AP,TRBD,CG13928,CG6654,MRE11,RAB27,TIO,TOM,TUTL,EN,TLD,HH,SOX15,SDR,E(SPL)MGAMMA-HLH,FNE,DLL,NUB,AL,DVE,E(SPL)M8-HLH,NAB,VMAT,NWK,DYSC,RN,TEY,DOC1,CG11438,DOPECR,E(SPL)MDELTA-HLH,VG,CCKLR-17D3,CG43347,PDM2,GABA-B-R3,E(SPL)M4-BFM,SMOG,BRD,WNT4,NMDAR1,WRY,CG10151,DOC2,GLUCLALPHA,DOP1R2,SCR,DAN,FD96CA,OCHO,NACHRBETA2,CNGL,NPF,NACHRALPHA1,SP1,GYC89DB,GL,LOV,BTD,OPTIX,SNPF,SCRT,TFAP-2,FD96CB,ATO,SIDPN,NACHRBETA1,DAC,NACHRALPHA5,CAS,NACHRALPHA6,TUT,TOY,DANR,VGLUT,NERFIN-1,HUG,ADA1-1 |
| GO:0007423 | sensory organ development | 1.6766E-06 | 1.6766E-06 | 1 | FT,SPI,EX,LAM,CHN,JUMU,BOI,DS,RL,CAUP,NEK2,FUTSCH,TSH,AB,TIO,TOM,TUTL,TLD,HH,SOX15,AL,E(SPL)M8-HLH,DYSC,RN,E(SPL)MDELTA-HLH,VG,E(SPL)M4-BFM,SCA,SCR,DAN,GL,OPTIX,ATO,DAC,TOY,DANR |
| GO:0035114 | imaginal disc-derived appendage morphogenesis | 1.6968E-06 | 1.6968E-06 | 1 | FT,SPI,DG,RHOGAP54D,JUMU,DS,RL,CAUP,TYN,TSH,TOK,NET,AP,EN,TLD,HH,DLL,AL,DVE,RN,VG,SCA,DAN,SP1,BTD,TFAP-2,ATO,DAC,DANR |
| GO:0048737 | imaginal disc-derived appendage development | 2.5955E-06 | 2.5955E-06 | 1 | FT,SPI,DG,RHOGAP54D,JUMU,DS,RL,CAUP,TYN,TSH,TOK,NET,AP,EN,TLD,HH,DLL,AL,DVE,RN,VG,SCA,DAN,SP1,BTD,TFAP-2,ATO,DAC,DANR |
| GO:0007444 | imaginal disc development | 4.2314E-06 | 4.2314E-06 | 1 | FT,SPI,DG,EX,RHOGAP54D,JUMU,BOI,DS,ESG,RL,CAUP,CKS30A,TYN,OTK,TSH,TOK,NET,AP,EN,TLD,HH,SOX15,E(SPL)MGAMMA-HLH,DLL,AL,DVE,NAB,RN,DOC1,VG,SCA,DAN,SP1,BTD,TFAP-2,ATO,DAC,TOY,DANR |
| GO:0048729 | tissue morphogenesis | 8.153E-06 | 8.153E-06 | 1 | FT,SPI,DG,TOLLO,LAM,PBL,RHOGAP54D,JUMU,EIP63E,SLI,DS,ESG,RL,CG17211,CAUP,CKS30A,TYN,OTK,TSH,TOK,NET,AP,TIO,EN,TLD,HH,MIPLE1,DLL,AL,DVE,RN,VG,SMOG,WNT4,SCA,SP1,BTD,TFAP-2,ATO,DAC |
| GO:0048666 | neuron development | 1.0303E-05 | 1.0303E-05 | 1 | DG,PAV,TOLLO,ROBO1,TOLL-7,CHN,PBL,STAI,BABOS,SLI,MUD,SLOW,DCP-1,MAS,FAX,SEMA2B,FUTSCH,OTK,AB,TEN-A,PDM3,TOK,AP,TUTL,EN,NRT,NUB,E(SPL)M8-HLH,NAB,DYSC,TEY,WNT4,ROBO3,HIG,DSCAM4,WRAPPER,OPTIX,ATO,DAC,NERFIN-1 |
| GO:0048749 | compound eye development | 1.0787E-05 | 1.0787E-05 | 1 | FT,SPI,EX,LAM,CHN,JUMU,BOI,DS,RL,CAUP,NEK2,FUTSCH,TSH,TIO,TUTL,TLD,HH,E(SPL)M8-HLH,DYSC,RN,E(SPL)MDELTA-HLH,VG,SCA,DAN,GL,OPTIX,ATO,DAC,TOY,DANR |
| GO:0065007 | biological regulation | 1.275E-05 | 1.275E-05 | 1 | BARK,FT,SPI,SSRP,DG,CG9135,PAV,YETI,EX,TOLLO,ROBO1,CG40191,TOLL-7,LAM,DCP2,CHN,PBL,KLP61F,STAI,INCENP,BABOS,POLO,CG1965,SMC2,CG9231,FZY,CG9125,RHOGAP54D,JUMU,BRAT,MARS,EIP63E,SLI,RAD50,BOI,DS,ESG,OLF413,RL,TEFU,STI,ROD,MSH6,RHOGEF4,CAUP,CG12299,CKS30A,CADPS,DCP-1,TYN,PIGS,L(3)NEO38,MAS,CG43366,NEK2,SEMA2B,FUTSCH,OTK,CG5466,TSH,AB,TEN-A,TET,PDM3,TOK,NET,AP,TRBD,CG13928,IA-2,CG6654,MRE11,RAB27,TIO,TOM,TUTL,MCO1,EN,TLD,HH,SOX15,SDR,E(SPL)MGAMMA-HLH,CG2650,FNE,CG7708,DLL,NUB,AL,DVE,E(SPL)M8-HLH,NAB,VMAT,NWK,DYSC,RN,NA,TEY,DOC1,CG11438,DOPECR,E(SPL)MDELTA-HLH,VG,MID1,CCKLR-17D3,CG43347,PDM2,GABA-B-R3,E(SPL)M4-BFM,SMOG,BRD,WNT4,NMDAR1,WRY,CG10151,DOC2,GLUCLALPHA,DOP1R2,SCR,CG3078,DAN,FD96CA,OCHO,TASK6,NACHRBETA2,CNGL,NPF,NACHRALPHA1,SP1,GYC89DB,GL,UNC-4,LOV,BTD,OPTIX,SNPF,SCRT,TFAP-2,FD96CB,ATO,SIDPN,NACHRBETA1,DAC,NACHRALPHA5,CAS,NACHRALPHA6,TUT,TOY,DANR,EIG71EE,VGLUT,NERFIN-1,HUG,ADA1-1 |
| GO:0000122 | negative regulation of transcription by RNA polymerase II | 1.7271E-05 | 1.7271E-05 | 1 | CHN,CG1965,ESG,CG12299,TSH,AB,NET,TIO,EN,SOX15,E(SPL)MGAMMA-HLH,DVE,E(SPL)M8-HLH,RN,DOC1,E(SPL)MDELTA-HLH,CG43347,DOC2,BTD,SCRT,TFAP-2,SIDPN,NERFIN-1 |
| GO:0008038 | neuron recognition | 2.1236E-05 | 2.1236E-05 | 1 | ROBO1,PBL,SLI,SLOW,SEMA2B,AB,TEN-A,TOK,TUTL,NRT,TEY,WNT4,ROBO3,HIG,DSCAM4,ATO |
| GO:0007389 | pattern specification process | 2.6193E-05 | 2.6193E-05 | 1 | FT,SPI,DG,ROBO1,DCP2,BRAT,SLI,BOI,DS,RL,CAUP,TSH,NET,AP,TIO,TOM,EN,TLD,HH,E(SPL)MGAMMA-HLH,DLL,DVE,E(SPL)M8-HLH,NAB,E(SPL)MDELTA-HLH,VG,SCA,SCR,DAN,BTD,TFAP-2,ATO,SIDPN,DAC,DANR |
| GO:0007560 | imaginal disc morphogenesis | 2.6584E-05 | 2.6584E-05 | 1 | FT,SPI,DG,RHOGAP54D,JUMU,DS,ESG,RL,CAUP,CKS30A,TYN,OTK,TSH,TOK,NET,AP,EN,TLD,HH,DLL,AL,DVE,RN,VG,SCA,SP1,BTD,TFAP-2,ATO,DAC |
| GO:0048563 | post-embryonic animal organ morphogenesis | 2.6584E-05 | 2.6584E-05 | 1 | FT,SPI,DG,RHOGAP54D,JUMU,DS,ESG,RL,CAUP,CKS30A,TYN,OTK,TSH,TOK,NET,AP,EN,TLD,HH,DLL,AL,DVE,RN,VG,SCA,SP1,BTD,TFAP-2,ATO,DAC |
| GO:0035218 | leg disc development | 2.7726E-05 | 2.7726E-05 | 1 | RHOGAP54D,DS,RL,TSH,AP,HH,DLL,AL,DVE,RN,VG,SP1,BTD,TFAP-2,DAC |
| GO:0060562 | epithelial tube morphogenesis | 3.1433E-05 | 3.1433E-05 | 1 | FT,SPI,DG,LAM,PBL,RHOGAP54D,JUMU,DS,ESG,RL,CG17211,CAUP,CKS30A,TYN,OTK,TSH,TOK,NET,AP,EN,TLD,HH,DLL,AL,DVE,RN,VG,SCA,SP1,BTD,TFAP-2,ATO,DAC |
| GO:0001654 | eye development | 3.3531E-05 | 3.3531E-05 | 1 | FT,SPI,EX,LAM,CHN,JUMU,BOI,DS,RL,CAUP,NEK2,FUTSCH,TSH,TIO,TUTL,TLD,HH,E(SPL)M8-HLH,DYSC,RN,E(SPL)MDELTA-HLH,VG,SCA,DAN,GL,OPTIX,ATO,DAC,TOY,DANR |
| GO:0048880 | sensory system development | 3.3531E-05 | 3.3531E-05 | 1 | FT,SPI,EX,LAM,CHN,JUMU,BOI,DS,RL,CAUP,NEK2,FUTSCH,TSH,TIO,TUTL,TLD,HH,E(SPL)M8-HLH,DYSC,RN,E(SPL)MDELTA-HLH,VG,SCA,DAN,GL,OPTIX,ATO,DAC,TOY,DANR |
| GO:0150063 | visual system development | 3.3531E-05 | 3.3531E-05 | 1 | FT,SPI,EX,LAM,CHN,JUMU,BOI,DS,RL,CAUP,NEK2,FUTSCH,TSH,TIO,TUTL,TLD,HH,E(SPL)M8-HLH,DYSC,RN,E(SPL)MDELTA-HLH,VG,SCA,DAN,GL,OPTIX,ATO,DAC,TOY,DANR |
| GO:0000902 | cell morphogenesis | 3.376E-05 | 3.376E-05 | 1 | DG,PAV,ROBO1,TOLL-7,CHN,PBL,BABOS,SLI,MUD,ESG,CKS30A,TYN,PIGS,FAX,SEMA2B,FUTSCH,OTK,AB,TEN-A,PDM3,TOK,AP,TUTL,EN,HH,NRT,NUB,E(SPL)M8-HLH,DYSC,VG,SMOG,WNT4,ROBO3,DSCAM4,OPTIX,DAC,NERFIN-1 |
| GO:0002009 | morphogenesis of an epithelium | 3.3973E-05 | 3.3973E-05 | 1 | FT,SPI,DG,TOLLO,LAM,PBL,RHOGAP54D,JUMU,EIP63E,DS,ESG,RL,CG17211,CAUP,CKS30A,TYN,OTK,TSH,TOK,NET,AP,TIO,EN,TLD,HH,DLL,AL,DVE,RN,VG,SMOG,WNT4,SCA,SP1,BTD,TFAP-2,ATO,DAC |
| GO:0008037 | cell recognition | 3.9415E-05 | 3.9415E-05 | 1 | ROBO1,PBL,SLI,SLOW,SEMA2B,AB,TEN-A,TOK,TUTL,NRT,TEY,WNT4,ROBO3,HIG,DSCAM4,ATO |
| GO:0003002 | regionalization | 4.5218E-05 | 4.5218E-05 | 1 | FT,DG,ROBO1,DCP2,BRAT,SLI,BOI,DS,RL,CAUP,TSH,NET,AP,TIO,TOM,EN,TLD,HH,E(SPL)MGAMMA-HLH,DLL,DVE,E(SPL)M8-HLH,NAB,E(SPL)MDELTA-HLH,VG,SCR,DAN,BTD,TFAP-2,ATO,SIDPN,DAC,DANR |
| GO:0048519 | negative regulation of biological process | 4.6855E-05 | 4.6855E-05 | 1 | FT,SPI,SSRP,DG,CG9135,PAV,EX,LAM,DCP2,CHN,PBL,POLO,CG1965,FZY,CG9125,BRAT,DS,ESG,RL,TEFU,STI,ROD,MSH6,CAUP,CG12299,DCP-1,PIGS,L(3)NEO38,CG43366,SEMA2B,FUTSCH,OTK,CG5466,TSH,AB,NET,TRBD,CG13928,MRE11,TIO,TOM,EN,HH,SOX15,SDR,E(SPL)MGAMMA-HLH,DLL,NUB,AL,DVE,E(SPL)M8-HLH,NWK,DYSC,RN,TEY,DOC1,E(SPL)MDELTA-HLH,CG43347,E(SPL)M4-BFM,BRD,DOC2,DOP1R2,OCHO,BTD,OPTIX,SCRT,TFAP-2,SIDPN,DAC,CAS,TUT,NERFIN-1 |
| GO:0031175 | neuron projection development | 4.7597E-05 | 4.7597E-05 | 1 | DG,PAV,ROBO1,TOLL-7,CHN,PBL,STAI,BABOS,SLI,MUD,MAS,FAX,SEMA2B,FUTSCH,OTK,AB,TEN-A,PDM3,TOK,AP,TUTL,EN,NRT,NUB,E(SPL)M8-HLH,DYSC,WNT4,ROBO3,DSCAM4,WRAPPER,OPTIX,DAC,NERFIN-1 |
| GO:0007417 | central nervous system development | 9.4221E-05 | 9.4221E-05 | 1 | LAM,CAP-D2,BRAT,MUD,ESG,STI,L(3)NEO38,MAS,AB,TEN-A,AP,EN,HH,NRT,DLL,PDM2,ROBO3,DAN,LOV,ATO,DAC,CAS,TOY,DANR |
| GO:0048523 | negative regulation of cellular process | 0.0001079 | 0.0001079 | 1 | FT,SPI,SSRP,PAV,EX,LAM,DCP2,CHN,PBL,POLO,CG1965,FZY,BRAT,DS,ESG,RL,TEFU,STI,ROD,MSH6,CG12299,DCP-1,PIGS,L(3)NEO38,CG43366,SEMA2B,FUTSCH,OTK,CG5466,TSH,AB,NET,TRBD,CG13928,MRE11,TIO,TOM,EN,HH,SOX15,SDR,E(SPL)MGAMMA-HLH,NUB,AL,DVE,E(SPL)M8-HLH,NWK,DYSC,RN,TEY,DOC1,E(SPL)MDELTA-HLH,CG43347,E(SPL)M4-BFM,BRD,DOC2,OCHO,BTD,OPTIX,SCRT,TFAP-2,SIDPN,CAS,TUT,NERFIN-1 |
| GO:0120039 | plasma membrane bounded cell projection morphogenesis | 0.00011055 | 0.00011055 | 1 | DG,PAV,ROBO1,TOLL-7,CHN,PBL,BABOS,SLI,MUD,FAX,SEMA2B,FUTSCH,OTK,AB,TEN-A,PDM3,TOK,AP,TUTL,EN,HH,NRT,NUB,E(SPL)M8-HLH,DYSC,WNT4,ROBO3,DSCAM4,OPTIX,DAC,NERFIN-1 |
| GO:0048858 | cell projection morphogenesis | 0.00011055 | 0.00011055 | 1 | DG,PAV,ROBO1,TOLL-7,CHN,PBL,BABOS,SLI,MUD,FAX,SEMA2B,FUTSCH,OTK,AB,TEN-A,PDM3,TOK,AP,TUTL,EN,HH,NRT,NUB,E(SPL)M8-HLH,DYSC,WNT4,ROBO3,DSCAM4,OPTIX,DAC,NERFIN-1 |
| GO:0045165 | cell fate commitment | 0.00011623 | 0.00011623 | 1 | SPI,PAV,CHN,POLO,BRAT,MUD,ESG,RL,PON,CAUP,AP,TOM,EN,TLD,HH,E(SPL)M8-HLH,RN,DOC1,E(SPL)M4-BFM,BRD,WNT4,DOC2,GL,ATO,DAC,CAS |
| GO:2000113 | negative regulation of cellular macromolecule biosynthetic process | 0.0001443 | 0.0001443 | 1 | LAM,DCP2,CHN,CG1965,BRAT,ESG,TEFU,CG12299,L(3)NEO38,CG5466,TSH,AB,NET,CG13928,TIO,EN,SOX15,E(SPL)MGAMMA-HLH,NUB,AL,DVE,E(SPL)M8-HLH,RN,TEY,DOC1,E(SPL)MDELTA-HLH,CG43347,DOC2,BTD,OPTIX,SCRT,TFAP-2,SIDPN,CAS,TUT,NERFIN-1 |
| GO:0010558 | negative regulation of macromolecule biosynthetic process | 0.0001443 | 0.0001443 | 1 | LAM,DCP2,CHN,CG1965,BRAT,ESG,TEFU,CG12299,L(3)NEO38,CG5466,TSH,AB,NET,CG13928,TIO,EN,SOX15,E(SPL)MGAMMA-HLH,NUB,AL,DVE,E(SPL)M8-HLH,RN,TEY,DOC1,E(SPL)MDELTA-HLH,CG43347,DOC2,BTD,OPTIX,SCRT,TFAP-2,SIDPN,CAS,TUT,NERFIN-1 |
| GO:0032990 | cell part morphogenesis | 0.00016611 | 0.00016611 | 1 | DG,PAV,ROBO1,TOLL-7,CHN,PBL,BABOS,SLI,MUD,FAX,SEMA2B,FUTSCH,OTK,AB,TEN-A,PDM3,TOK,AP,TUTL,EN,HH,NRT,NUB,E(SPL)M8-HLH,DYSC,WNT4,ROBO3,DSCAM4,OPTIX,DAC,NERFIN-1 |
| GO:0000904 | cell morphogenesis involved in differentiation | 0.00021104 | 0.00021104 | 1 | DG,ROBO1,TOLL-7,CHN,PBL,BABOS,SLI,MUD,TYN,PIGS,FAX,SEMA2B,FUTSCH,OTK,AB,TEN-A,PDM3,TOK,AP,TUTL,EN,NRT,NUB,E(SPL)M8-HLH,DYSC,SMOG,WNT4,ROBO3,DSCAM4,OPTIX,DAC,NERFIN-1 |
| GO:0007449 | proximal/distal pattern formation, imaginal disc | 0.00021487 | 0.00021487 | 1 | RL,TSH,AP,HH,DLL,VG,DAC |
| GO:0120036 | plasma membrane bounded cell projection organization | 0.0002213 | 0.0002213 | 1 | FT,DG,PAV,ROBO1,TOLL-7,CHN,PBL,STAI,BABOS,SLI,MUD,DS,ANA3,MAS,FAX,SEMA2B,FUTSCH,OTK,AB,TEN-A,PDM3,TOK,AP,TUTL,EN,CG5142,HH,NRT,NUB,E(SPL)M8-HLH,DYSC,WNT4,ROBO3,CG17083,BBS8,DSCAM4,WRAPPER,OPTIX,DAC,NERFIN-1 |
| GO:0061564 | axon development | 0.00022504 | 0.00022504 | 1 | DG,ROBO1,TOLL-7,PBL,SLI,MUD,MAS,FAX,SEMA2B,FUTSCH,OTK,AB,TEN-A,PDM3,TOK,AP,TUTL,EN,NRT,DYSC,WNT4,ROBO3,DSCAM4,WRAPPER,DAC,NERFIN-1 |
| GO:0045892 | negative regulation of transcription, DNA-templated | 0.0002322 | 0.0002322 | 1 | LAM,CHN,CG1965,ESG,TEFU,CG12299,L(3)NEO38,CG5466,TSH,AB,NET,TIO,EN,SOX15,E(SPL)MGAMMA-HLH,NUB,AL,DVE,E(SPL)M8-HLH,RN,TEY,DOC1,E(SPL)MDELTA-HLH,CG43347,DOC2,BTD,OPTIX,SCRT,TFAP-2,SIDPN,CAS,NERFIN-1 |
| GO:1903507 | negative regulation of nucleic acid-templated transcription | 0.0002322 | 0.0002322 | 1 | LAM,CHN,CG1965,ESG,TEFU,CG12299,L(3)NEO38,CG5466,TSH,AB,NET,TIO,EN,SOX15,E(SPL)MGAMMA-HLH,NUB,AL,DVE,E(SPL)M8-HLH,RN,TEY,DOC1,E(SPL)MDELTA-HLH,CG43347,DOC2,BTD,OPTIX,SCRT,TFAP-2,SIDPN,CAS,NERFIN-1 |
| GO:1902679 | negative regulation of RNA biosynthetic process | 0.0002322 | 0.0002322 | 1 | LAM,CHN,CG1965,ESG,TEFU,CG12299,L(3)NEO38,CG5466,TSH,AB,NET,TIO,EN,SOX15,E(SPL)MGAMMA-HLH,NUB,AL,DVE,E(SPL)M8-HLH,RN,TEY,DOC1,E(SPL)MDELTA-HLH,CG43347,DOC2,BTD,OPTIX,SCRT,TFAP-2,SIDPN,CAS,NERFIN-1 |
| GO:0035120 | post-embryonic appendage morphogenesis | 0.00025574 | 0.00025574 | 1 | FT,SPI,DG,RHOGAP54D,JUMU,DS,RL,CAUP,TYN,TSH,TOK,NET,AP,EN,TLD,HH,DLL,AL,DVE,RN,VG,SCA,SP1,BTD,TFAP-2 |
| GO:0035239 | tube morphogenesis | 0.00025982 | 0.00025982 | 1 | FT,SPI,DG,LAM,PBL,RHOGAP54D,JUMU,DS,ESG,RL,CG17211,CAUP,CKS30A,TYN,OTK,TSH,TOK,NET,AP,EN,TLD,HH,DLL,AL,DVE,RN,VG,SCA,SP1,BTD,TFAP-2,ATO,DAC |
| GO:0008039 | synaptic target recognition | 0.00026382 | 0.00026382 | 1 | SLI,SLOW,SEMA2B,AB,TEN-A,TUTL,TEY,WNT4,ROBO3,HIG |
| GO:0031327 | negative regulation of cellular biosynthetic process | 0.00027541 | 0.00027541 | 1 | LAM,DCP2,CHN,CG1965,BRAT,ESG,TEFU,CG12299,L(3)NEO38,CG5466,TSH,AB,NET,CG13928,TIO,EN,SOX15,E(SPL)MGAMMA-HLH,NUB,AL,DVE,E(SPL)M8-HLH,RN,TEY,DOC1,E(SPL)MDELTA-HLH,CG43347,DOC2,BTD,OPTIX,SCRT,TFAP-2,SIDPN,CAS,TUT,NERFIN-1 |
| GO:0060322 | head development | 0.00028588 | 0.00028588 | 1 | BRAT,MUD,L(3)NEO38,TSH,AB,TEN-A,TIO,EN,HH,DLL,ROBO3,BTD,ATO,DAC,CAS,TOY |
| GO:0010605 | negative regulation of macromolecule metabolic process | 0.00028942 | 0.00028942 | 1 | FT,SPI,SSRP,DG,LAM,DCP2,CHN,CG1965,CG9125,BRAT,ESG,TEFU,MSH6,CG12299,L(3)NEO38,CG43366,CG5466,TSH,AB,NET,CG13928,TIO,EN,HH,SOX15,E(SPL)MGAMMA-HLH,DLL,NUB,AL,DVE,E(SPL)M8-HLH,RN,TEY,DOC1,E(SPL)MDELTA-HLH,CG43347,DOC2,BTD,OPTIX,SCRT,TFAP-2,SIDPN,DAC,CAS,TUT,NERFIN-1 |
| GO:0045934 | negative regulation of nucleobase-containing compound metabolic process | 0.00031184 | 0.00031184 | 1 | LAM,CHN,CG1965,ESG,TEFU,MSH6,CG12299,L(3)NEO38,CG5466,TSH,AB,NET,TIO,EN,SOX15,E(SPL)MGAMMA-HLH,NUB,AL,DVE,E(SPL)M8-HLH,RN,TEY,DOC1,E(SPL)MDELTA-HLH,CG43347,DOC2,BTD,OPTIX,SCRT,TFAP-2,SIDPN,CAS,NERFIN-1 |
| GO:0009890 | negative regulation of biosynthetic process | 0.00031244 | 0.00031244 | 1 | LAM,DCP2,CHN,CG1965,BRAT,ESG,TEFU,CG12299,L(3)NEO38,CG5466,TSH,AB,NET,CG13928,TIO,EN,SOX15,E(SPL)MGAMMA-HLH,NUB,AL,DVE,E(SPL)M8-HLH,RN,TEY,DOC1,E(SPL)MDELTA-HLH,CG43347,DOC2,BTD,OPTIX,SCRT,TFAP-2,SIDPN,CAS,TUT,NERFIN-1 |
| GO:0048812 | neuron projection morphogenesis | 0.00033275 | 0.00033275 | 1 | DG,PAV,ROBO1,TOLL-7,CHN,PBL,BABOS,SLI,MUD,FAX,SEMA2B,FUTSCH,OTK,AB,TEN-A,PDM3,TOK,AP,TUTL,EN,NRT,NUB,E(SPL)M8-HLH,DYSC,WNT4,ROBO3,DSCAM4,OPTIX,DAC,NERFIN-1 |
| GO:0048707 | instar larval or pupal morphogenesis | 0.00034958 | 0.00034958 | 1 | FT,SPI,DG,RHOGAP54D,JUMU,DS,ESG,RL,CAUP,CKS30A,TYN,OTK,TSH,TOK,NET,AP,EN,TLD,HH,DLL,AL,DVE,RN,VG,SCA,SP1,BTD,TFAP-2,ATO,DAC |
| GO:0030030 | cell projection organization | 0.0003509 | 0.0003509 | 1 | FT,DG,PAV,ROBO1,TOLL-7,CHN,PBL,STAI,BABOS,SLI,MUD,DS,ANA3,MAS,FAX,SEMA2B,FUTSCH,OTK,AB,TEN-A,PDM3,TOK,AP,TUTL,EN,CG5142,HH,NRT,NUB,E(SPL)M8-HLH,DYSC,WNT4,ROBO3,CG17083,BBS8,DSCAM4,WRAPPER,OPTIX,DAC,NERFIN-1 |
| GO:0007552 | metamorphosis | 0.00038088 | 0.00038088 | 1 | FT,SPI,DG,RHOGAP54D,JUMU,EIP63E,DS,ESG,RL,CAUP,CKS30A,TYN,OTK,TSH,TOK,NET,AP,EN,TLD,HH,DLL,AL,DVE,RN,VG,SCA,SP1,BTD,TFAP-2,ATO,DAC |
| GO:0006351 | transcription, DNA-templated | 0.00039985 | 0.00039985 | 1 | LAM,CHN,RPII140,CG1965,JUMU,BRAT,EIP63E,ESG,RL,TEFU,CAUP,CG12299,RPB12,L(3)NEO38,CG5466,TSH,AB,TET,PDM3,NET,AP,CG6654,TIO,EN,SOX15,E(SPL)MGAMMA-HLH,DLL,NUB,AL,DVE,E(SPL)M8-HLH,NAB,RN,TEY,DOC1,E(SPL)MDELTA-HLH,VG,CG43347,PDM2,CG10151,DOC2,SCR,DAN,FD96CA,SP1,GL,LOV,BTD,OPTIX,SCRT,TFAP-2,FD96CB,ATO,SIDPN,DAC,CAS,TOY,DANR,NERFIN-1,ADA1-1 |
| GO:0097659 | nucleic acid-templated transcription | 0.00039985 | 0.00039985 | 1 | LAM,CHN,RPII140,CG1965,JUMU,BRAT,EIP63E,ESG,RL,TEFU,CAUP,CG12299,RPB12,L(3)NEO38,CG5466,TSH,AB,TET,PDM3,NET,AP,CG6654,TIO,EN,SOX15,E(SPL)MGAMMA-HLH,DLL,NUB,AL,DVE,E(SPL)M8-HLH,NAB,RN,TEY,DOC1,E(SPL)MDELTA-HLH,VG,CG43347,PDM2,CG10151,DOC2,SCR,DAN,FD96CA,SP1,GL,LOV,BTD,OPTIX,SCRT,TFAP-2,FD96CB,ATO,SIDPN,DAC,CAS,TOY,DANR,NERFIN-1,ADA1-1 |
| GO:0032774 | RNA biosynthetic process | 0.00042214 | 0.00042214 | 1 | LAM,CHN,RPII140,CG1965,JUMU,BRAT,EIP63E,ESG,RL,TEFU,CAUP,CG12299,RPB12,L(3)NEO38,CG5466,TSH,AB,TET,PDM3,NET,AP,CG6654,TIO,EN,SOX15,E(SPL)MGAMMA-HLH,DLL,NUB,AL,DVE,E(SPL)M8-HLH,NAB,RN,TEY,DOC1,E(SPL)MDELTA-HLH,VG,CG43347,PDM2,CG10151,DOC2,SCR,DAN,FD96CA,SP1,GL,LOV,BTD,OPTIX,SCRT,TFAP-2,FD96CB,ATO,SIDPN,DAC,CAS,TOY,DANR,NERFIN-1,ADA1-1 |
| GO:0048468 | cell development | 0.00045325 | 0.00045325 | 1 | SPI,SSRP,DG,PAV,EX,TOLLO,ROBO1,TOLL-7,DCP2,CHN,PBL,STAI,BABOS,POLO,MCM6,BRAT,SLI,MUD,RL,TEFU,CAUP,SLOW,DCP-1,TYN,PIGS,MAS,FAX,SEMA2B,FUTSCH,OTK,AB,TEN-A,TET,PDM3,TOK,AP,TUTL,EN,TLD,HH,E(SPL)MGAMMA-HLH,NRT,NUB,E(SPL)M8-HLH,NAB,DYSC,TEY,E(SPL)MDELTA-HLH,PDM2,SMOG,WNT4,ROBO3,HIG,DSCAM4,WRAPPER,LOV,OPTIX,ATO,SIDPN,DAC,CAS,TUT,NERFIN-1 |
| GO:0051172 | negative regulation of nitrogen compound metabolic process | 0.00049163 | 0.00049163 | 1 | SSRP,LAM,DCP2,CHN,CG1965,BRAT,ESG,TEFU,MSH6,CG12299,L(3)NEO38,CG43366,CG5466,TSH,AB,NET,CG13928,TIO,EN,HH,SOX15,E(SPL)MGAMMA-HLH,NUB,AL,DVE,E(SPL)M8-HLH,RN,TEY,DOC1,E(SPL)MDELTA-HLH,CG43347,DOC2,BTD,OPTIX,SCRT,TFAP-2,SIDPN,CAS,TUT,NERFIN-1 |
| GO:0051253 | negative regulation of RNA metabolic process | 0.00055871 | 0.00055871 | 1 | LAM,CHN,CG1965,ESG,TEFU,CG12299,L(3)NEO38,CG5466,TSH,AB,NET,TIO,EN,SOX15,E(SPL)MGAMMA-HLH,NUB,AL,DVE,E(SPL)M8-HLH,RN,TEY,DOC1,E(SPL)MDELTA-HLH,CG43347,DOC2,BTD,OPTIX,SCRT,TFAP-2,SIDPN,CAS,NERFIN-1 |
| GO:0007478 | leg disc morphogenesis | 0.00056985 | 0.00056985 | 1 | RHOGAP54D,DS,AP,HH,DLL,AL,DVE,RN,VG,SP1,BTD,TFAP-2 |
| GO:0048859 | formation of anatomical boundary | 0.00057397 | 0.00057397 | 1 | FT,ROBO1,SLI,DS,CAUP,TSH,TOM,E(SPL)M8-HLH,SCR,ATO |
| GO:0009892 | negative regulation of metabolic process | 0.0005935 | 0.0005935 | 1 | FT,SPI,SSRP,DG,LAM,DCP2,CHN,CG1965,CG9125,BRAT,ESG,RL,TEFU,MSH6,CG12299,L(3)NEO38,CG43366,CG5466,TSH,AB,NET,CG13928,TIO,EN,HH,SOX15,E(SPL)MGAMMA-HLH,DLL,NUB,AL,DVE,E(SPL)M8-HLH,RN,TEY,DOC1,E(SPL)MDELTA-HLH,CG43347,DOC2,BTD,OPTIX,SCRT,TFAP-2,SIDPN,DAC,CAS,TUT,NERFIN-1 |
| GO:0042127 | regulation of cell population proliferation | 0.00068684 | 0.00068684 | 1 | FT,SPI,EX,LAM,POLO,BRAT,ESG,RL,TSH,HH,SOX15,E(SPL)MGAMMA-HLH,NUB,E(SPL)MDELTA-HLH,VG,PDM2,WNT4,BTD,TFAP-2,CAS |
| GO:0006355 | regulation of transcription, DNA-templated | 0.00069465 | 0.00069465 | 1 | LAM,CHN,CG1965,JUMU,EIP63E,ESG,RL,TEFU,CAUP,CG12299,L(3)NEO38,CG5466,TSH,AB,TET,PDM3,NET,AP,CG6654,TIO,EN,SOX15,E(SPL)MGAMMA-HLH,DLL,NUB,AL,DVE,E(SPL)M8-HLH,NAB,RN,TEY,DOC1,E(SPL)MDELTA-HLH,VG,CG43347,PDM2,CG10151,DOC2,SCR,DAN,FD96CA,SP1,GL,LOV,BTD,OPTIX,SCRT,TFAP-2,FD96CB,ATO,SIDPN,DAC,CAS,TOY,DANR,NERFIN-1,ADA1-1 |
| GO:1903506 | regulation of nucleic acid-templated transcription | 0.00069465 | 0.00069465 | 1 | LAM,CHN,CG1965,JUMU,EIP63E,ESG,RL,TEFU,CAUP,CG12299,L(3)NEO38,CG5466,TSH,AB,TET,PDM3,NET,AP,CG6654,TIO,EN,SOX15,E(SPL)MGAMMA-HLH,DLL,NUB,AL,DVE,E(SPL)M8-HLH,NAB,RN,TEY,DOC1,E(SPL)MDELTA-HLH,VG,CG43347,PDM2,CG10151,DOC2,SCR,DAN,FD96CA,SP1,GL,LOV,BTD,OPTIX,SCRT,TFAP-2,FD96CB,ATO,SIDPN,DAC,CAS,TOY,DANR,NERFIN-1,ADA1-1 |
| GO:2001141 | regulation of RNA biosynthetic process | 0.00069465 | 0.00069465 | 1 | LAM,CHN,CG1965,JUMU,EIP63E,ESG,RL,TEFU,CAUP,CG12299,L(3)NEO38,CG5466,TSH,AB,TET,PDM3,NET,AP,CG6654,TIO,EN,SOX15,E(SPL)MGAMMA-HLH,DLL,NUB,AL,DVE,E(SPL)M8-HLH,NAB,RN,TEY,DOC1,E(SPL)MDELTA-HLH,VG,CG43347,PDM2,CG10151,DOC2,SCR,DAN,FD96CA,SP1,GL,LOV,BTD,OPTIX,SCRT,TFAP-2,FD96CB,ATO,SIDPN,DAC,CAS,TOY,DANR,NERFIN-1,ADA1-1 |
| GO:0048569 | post-embryonic animal organ development | 0.0006967 | 0.0006967 | 1 | FT,SPI,DG,CHN,RHOGAP54D,JUMU,DS,ESG,RL,CAUP,CKS30A,TYN,OTK,TSH,TOK,NET,AP,EN,TLD,HH,DLL,AL,DVE,RN,VG,SCA,SP1,BTD,TFAP-2,ATO,DAC |
| GO:0009886 | post-embryonic animal morphogenesis | 0.00071829 | 0.00071829 | 1 | FT,SPI,DG,RHOGAP54D,JUMU,DS,ESG,RL,CAUP,CKS30A,TYN,OTK,TSH,TOK,NET,AP,EN,TLD,HH,DLL,AL,DVE,RN,VG,SCA,SP1,BTD,TFAP-2,ATO,DAC |
| GO:0048667 | cell morphogenesis involved in neuron differentiation | 0.00084373 | 0.00084373 | 1 | DG,ROBO1,TOLL-7,CHN,PBL,BABOS,SLI,MUD,FAX,SEMA2B,FUTSCH,OTK,AB,TEN-A,PDM3,TOK,AP,TUTL,EN,NRT,NUB,E(SPL)M8-HLH,DYSC,WNT4,ROBO3,DSCAM4,OPTIX,DAC,NERFIN-1 |
| GO:0042330 | taxis | 0.0009604 | 0.0009604 | 1 | DG,ROBO1,TOLL-7,SLI,MUD,ESG,SEMA2B,OTK,AB,TEN-A,PDM3,TOK,AP,TUTL,EN,HH,NRT,DYSC,WNT4,NMDAR1,ROBO3,DSCAM4,LOV,DAC,NERFIN-1 |
| GO:2000177 | regulation of neural precursor cell proliferation | 0.00137816 | 0.00137816 | 1 | POLO,BRAT,ESG,HH,E(SPL)MGAMMA-HLH,NUB,E(SPL)MDELTA-HLH,PDM2,BTD,CAS |
| GO:0006935 | chemotaxis | 0.00138117 | 0.00138117 | 1 | DG,ROBO1,TOLL-7,SLI,MUD,SEMA2B,OTK,AB,TEN-A,PDM3,TOK,AP,TUTL,EN,HH,NRT,DYSC,WNT4,ROBO3,DSCAM4,DAC,NERFIN-1 |
| GO:0007409 | axonogenesis | 0.00140123 | 0.00140123 | 1 | DG,ROBO1,TOLL-7,PBL,SLI,MUD,FAX,SEMA2B,FUTSCH,OTK,AB,TEN-A,PDM3,TOK,AP,TUTL,EN,NRT,DYSC,WNT4,ROBO3,DSCAM4,DAC,NERFIN-1 |
| GO:0006366 | transcription by RNA polymerase II | 0.00155648 | 0.00155648 | 1 | CHN,RPII140,CG1965,ESG,CAUP,CG12299,RPB12,L(3)NEO38,TSH,AB,TET,PDM3,NET,AP,CG6654,TIO,EN,SOX15,E(SPL)MGAMMA-HLH,DLL,NUB,AL,DVE,E(SPL)M8-HLH,RN,DOC1,E(SPL)MDELTA-HLH,VG,CG43347,PDM2,DOC2,SCR,FD96CA,SP1,GL,LOV,BTD,OPTIX,SCRT,TFAP-2,FD96CB,ATO,SIDPN,DAC,TOY,NERFIN-1,ADA1-1 |
| GO:0031324 | negative regulation of cellular metabolic process | 0.00166136 | 0.00166136 | 1 | SSRP,LAM,DCP2,CHN,CG1965,BRAT,ESG,RL,TEFU,MSH6,CG12299,L(3)NEO38,CG43366,CG5466,TSH,AB,NET,CG13928,TIO,EN,HH,SOX15,E(SPL)MGAMMA-HLH,NUB,AL,DVE,E(SPL)M8-HLH,RN,TEY,DOC1,E(SPL)MDELTA-HLH,CG43347,DOC2,BTD,OPTIX,SCRT,TFAP-2,SIDPN,CAS,TUT,NERFIN-1 |
| GO:2000112 | regulation of cellular macromolecule biosynthetic process | 0.00177805 | 0.00177805 | 1 | TOLLO,LAM,DCP2,CHN,CG1965,JUMU,BRAT,EIP63E,ESG,RL,TEFU,CAUP,CG12299,L(3)NEO38,CG5466,TSH,AB,TET,PDM3,NET,AP,CG13928,CG6654,TIO,EN,SOX15,E(SPL)MGAMMA-HLH,DLL,NUB,AL,DVE,E(SPL)M8-HLH,NAB,RN,TEY,DOC1,E(SPL)MDELTA-HLH,VG,CG43347,PDM2,CG10151,DOC2,SCR,DAN,FD96CA,SP1,GL,LOV,BTD,OPTIX,SCRT,TFAP-2,FD96CB,ATO,SIDPN,DAC,CAS,TUT,TOY,DANR,NERFIN-1,ADA1-1 |
| GO:0007267 | cell-cell signaling | 0.00183006 | 0.00183006 | 1 | FT,DG,PAV,PBL,EIP63E,DS,RL,CADPS,DCP-1,NEK2,OTK,TSH,TEN-A,TRBD,HH,VACHT,NWK,DYSC,VG,GABA-B-R3,WNT4,NMDAR1,GLUCLALPHA,CSAS,NACHRBETA2,NPF,NACHRALPHA1,NACHRBETA1,NACHRALPHA5,NACHRALPHA6,VGLUT |
| GO:0007411 | axon guidance | 0.00185389 | 0.00185389 | 1 | DG,ROBO1,TOLL-7,SLI,MUD,SEMA2B,OTK,AB,TEN-A,PDM3,TOK,AP,TUTL,EN,NRT,DYSC,WNT4,ROBO3,DSCAM4,DAC,NERFIN-1 |
| GO:0051252 | regulation of RNA metabolic process | 0.00198176 | 0.00198176 | 1 | DG,LAM,DCP2,CHN,CG1965,JUMU,EIP63E,ESG,RL,TEFU,CAUP,CG12299,L(3)NEO38,CG5466,TSH,AB,TET,PDM3,NET,AP,CG13928,CG6654,TIO,EN,SOX15,E(SPL)MGAMMA-HLH,FNE,DLL,NUB,AL,DVE,E(SPL)M8-HLH,NAB,RN,TEY,DOC1,E(SPL)MDELTA-HLH,VG,CG43347,PDM2,CG10151,DOC2,SCR,DAN,FD96CA,SP1,GL,LOV,BTD,OPTIX,SCRT,TFAP-2,FD96CB,ATO,SIDPN,DAC,CAS,TOY,DANR,NERFIN-1,ADA1-1 |
| GO:0010556 | regulation of macromolecule biosynthetic process | 0.00200755 | 0.00200755 | 1 | TOLLO,LAM,DCP2,CHN,CG1965,JUMU,BRAT,EIP63E,ESG,RL,TEFU,CAUP,CG12299,L(3)NEO38,CG5466,TSH,AB,TET,PDM3,NET,AP,CG13928,CG6654,TIO,EN,SOX15,E(SPL)MGAMMA-HLH,DLL,NUB,AL,DVE,E(SPL)M8-HLH,NAB,RN,TEY,DOC1,E(SPL)MDELTA-HLH,VG,CG43347,PDM2,CG10151,DOC2,SCR,DAN,FD96CA,SP1,GL,LOV,BTD,OPTIX,SCRT,TFAP-2,FD96CB,ATO,SIDPN,DAC,CAS,TUT,TOY,DANR,NERFIN-1,ADA1-1 |
| GO:0006357 | regulation of transcription by RNA polymerase II | 0.00210507 | 0.00210507 | 1 | CHN,CG1965,ESG,CAUP,CG12299,L(3)NEO38,TSH,AB,TET,PDM3,NET,AP,CG6654,TIO,EN,SOX15,E(SPL)MGAMMA-HLH,DLL,NUB,AL,DVE,E(SPL)M8-HLH,RN,DOC1,E(SPL)MDELTA-HLH,VG,CG43347,PDM2,DOC2,SCR,FD96CA,SP1,GL,LOV,BTD,OPTIX,SCRT,TFAP-2,FD96CB,ATO,SIDPN,DAC,TOY,NERFIN-1,ADA1-1 |
| GO:0009954 | proximal/distal pattern formation | 0.00241961 | 0.00241961 | 1 | RL,TSH,AP,HH,DLL,VG,DAC |
| GO:0007480 | imaginal disc-derived leg morphogenesis | 0.00246285 | 0.00246285 | 1 | RHOGAP54D,DS,AP,DLL,AL,DVE,RN,VG,SP1,BTD,TFAP-2 |
| GO:0007049 | cell cycle | 0.00247483 | 0.00247483 | 1 | PAV,YETI,EX,MCM2,CG40191,LAM,PBL,KLP61F,INCENP,DNAPOL-ALPHA180,POLO,SPD-2,MCM6,CAP-D2,MINK,SMC2,FZY,CG12018,KLP67A,MARS,EIP63E,RAD50,MUD,ESG,SPC105R,RL,TEFU,STI,ROD,MSH6,CAUP,ANA3,CKS30A,NEK2,CG5466,MRE11,HH,VG,COROLLA,NERFIN-1 |
| GO:0019219 | regulation of nucleobase-containing compound metabolic process | 0.00249237 | 0.00249237 | 1 | DG,LAM,DCP2,CHN,CG1965,JUMU,EIP63E,ESG,RL,TEFU,MSH6,CAUP,CG12299,L(3)NEO38,CG5466,TSH,AB,TET,PDM3,NET,AP,CG13928,CG6654,TIO,EN,SOX15,E(SPL)MGAMMA-HLH,FNE,DLL,NUB,AL,DVE,E(SPL)M8-HLH,NAB,RN,TEY,DOC1,E(SPL)MDELTA-HLH,VG,CG43347,PDM2,CG10151,DOC2,SCR,DAN,FD96CA,SP1,GL,LOV,BTD,OPTIX,SCRT,TFAP-2,FD96CB,ATO,SIDPN,DAC,CAS,TOY,DANR,NERFIN-1,ADA1-1 |
| GO:0097485 | neuron projection guidance | 0.00301169 | 0.00301169 | 1 | DG,ROBO1,TOLL-7,SLI,MUD,SEMA2B,OTK,AB,TEN-A,PDM3,TOK,AP,TUTL,EN,NRT,DYSC,WNT4,ROBO3,DSCAM4,DAC,NERFIN-1 |
| GO:0031326 | regulation of cellular biosynthetic process | 0.00324722 | 0.00324722 | 1 | TOLLO,LAM,DCP2,CHN,CG1965,JUMU,BRAT,EIP63E,ESG,RL,TEFU,CAUP,CG12299,L(3)NEO38,CG5466,TSH,AB,TET,PDM3,NET,AP,CG13928,CG6654,TIO,EN,SOX15,E(SPL)MGAMMA-HLH,DLL,NUB,AL,DVE,E(SPL)M8-HLH,NAB,RN,TEY,DOC1,E(SPL)MDELTA-HLH,VG,CG43347,PDM2,SMOG,CG10151,DOC2,SCR,DAN,FD96CA,SP1,GL,LOV,BTD,OPTIX,SCRT,TFAP-2,FD96CB,ATO,SIDPN,DAC,CAS,TUT,TOY,DANR,NERFIN-1,ADA1-1 |
| GO:0000278 | mitotic cell cycle | 0.00336881 | 0.00336881 | 1 | PAV,YETI,EX,MCM2,LAM,PBL,KLP61F,INCENP,DNAPOL-ALPHA180,POLO,MCM6,CAP-D2,MINK,SMC2,FZY,CG12018,KLP67A,MARS,EIP63E,MUD,RL,TEFU,STI,ROD,CAUP,CKS30A,NEK2,MRE11,HH,VG |
| GO:0007447 | imaginal disc pattern formation | 0.00344785 | 0.00344785 | 1 | FT,BOI,DS,RL,TSH,AP,EN,HH,E(SPL)MGAMMA-HLH,DLL,NAB,VG,DAC |
| GO:0022610 | biological adhesion | 0.00370735 | 0.00370735 | 1 | FT,ROBO1,NIJA,SLI,DS,TYN,SEMA2B,OTK,TEN-A,TUTL,HH,NRT,CG17716,DSCAM4,SGS7,WRAPPER,EIG71EE,SGS3,SGS5 |
| GO:0009889 | regulation of biosynthetic process | 0.00381775 | 0.00381775 | 1 | TOLLO,LAM,DCP2,CHN,CG1965,JUMU,BRAT,EIP63E,ESG,RL,TEFU,CAUP,CG12299,L(3)NEO38,CG5466,TSH,AB,TET,PDM3,NET,AP,CG13928,CG6654,TIO,EN,SOX15,E(SPL)MGAMMA-HLH,DLL,NUB,AL,DVE,E(SPL)M8-HLH,NAB,RN,TEY,DOC1,E(SPL)MDELTA-HLH,VG,CG43347,PDM2,SMOG,CG10151,DOC2,SCR,DAN,FD96CA,SP1,GL,LOV,BTD,OPTIX,SCRT,TFAP-2,FD96CB,ATO,SIDPN,DAC,CAS,TUT,TOY,DANR,NERFIN-1,ADA1-1 |
| GO:0032989 | cellular component morphogenesis | 0.00454535 | 0.00454535 | 1 | DG,PAV,ROBO1,TOLL-7,CHN,PBL,BABOS,MCM6,SLI,MUD,RL,FAX,SEMA2B,FUTSCH,OTK,AB,TEN-A,PDM3,TOK,AP,TUTL,EN,HH,NRT,NUB,E(SPL)M8-HLH,DYSC,WNT4,ROBO3,DSCAM4,OPTIX,DAC,NERFIN-1 |
| GO:1903047 | mitotic cell cycle process | 0.00469091 | 0.00469091 | 1 | PAV,YETI,EX,MCM2,LAM,PBL,KLP61F,INCENP,DNAPOL-ALPHA180,POLO,MCM6,CAP-D2,MINK,FZY,CG12018,KLP67A,MARS,EIP63E,MUD,TEFU,STI,ROD,NEK2,MRE11 |
| GO:0048608 | reproductive structure development | 0.00528454 | 0.00528454 | 1 | ROBO1,LAM,SLI,OTK,EN,HH,DLL,DVE,WNT4,ROBO3,TFAP-2 |
| GO:0061458 | reproductive system development | 0.00528454 | 0.00528454 | 1 | ROBO1,LAM,SLI,OTK,EN,HH,DLL,DVE,WNT4,ROBO3,TFAP-2 |
| GO:0022402 | cell cycle process | 0.00529482 | 0.00529482 | 1 | PAV,YETI,EX,MCM2,LAM,PBL,KLP61F,INCENP,DNAPOL-ALPHA180,POLO,SPD-2,MCM6,CAP-D2,MINK,FZY,CG12018,KLP67A,MARS,EIP63E,RAD50,MUD,ESG,SPC105R,TEFU,STI,ROD,MSH6,ANA3,CKS30A,NEK2,CG5466,MRE11,COROLLA,NERFIN-1 |
| GO:0035161 | imaginal disc lineage restriction | 0.00562388 | 0.00562388 | 1 | AP,EN,HH,VG |
| GO:0002165 | instar larval or pupal development | 0.00604941 | 0.00604941 | 1 | FT,SPI,DG,RHOGAP54D,JUMU,EIP63E,DS,ESG,RL,CAUP,CKS30A,TYN,OTK,TSH,TOK,NET,AP,EN,TLD,HH,DLL,AL,DVE,RN,VG,LCP9,SCA,SP1,BTD,TFAP-2,ATO,DAC |
| GO:0048645 | animal organ formation | 0.0063425 | 0.0063425 | 1 | ROBO1,SLI,TSH,TOM,HH,DLL,E(SPL)M8-HLH,SCR,ATO |
| GO:0010468 | regulation of gene expression | 0.00649116 | 0.00649116 | 1 | FT,SPI,DG,TOLL-7,LAM,DCP2,CHN,CG1965,CG9125,JUMU,BRAT,EIP63E,ESG,RL,TEFU,CAUP,CG12299,L(3)NEO38,CG5466,TSH,AB,TET,PDM3,NET,AP,CG13928,CG6654,TIO,EN,HH,SOX15,E(SPL)MGAMMA-HLH,DLL,NUB,AL,DVE,E(SPL)M8-HLH,NAB,RN,TEY,DOC1,E(SPL)MDELTA-HLH,VG,CG43347,PDM2,SMOG,CG10151,DOC2,SCR,DAN,FD96CA,SP1,GL,UNC-4,LOV,BTD,OPTIX,SCRT,TFAP-2,FD96CB,ATO,SIDPN,DAC,CAS,TUT,TOY,DANR,NERFIN-1,ADA1-1 |
| GO:0035220 | wing disc development | 0.006859 | 0.006859 | 1 | FT,SPI,DG,JUMU,BOI,DS,ESG,RL,CAUP,TYN,TSH,TOK,NET,AP,EN,TLD,HH,SOX15,E(SPL)MGAMMA-HLH,DLL,DVE,NAB,DOC1,VG,SCA |
| GO:0061351 | neural precursor cell proliferation | 0.00712609 | 0.00712609 | 1 | POLO,BRAT,MUD,ESG,PON,HH,E(SPL)MGAMMA-HLH,NUB,E(SPL)MDELTA-HLH,PDM2,BTD,CAS |
| GO:0001745 | compound eye morphogenesis | 0.00745259 | 0.00745259 | 1 | FT,SPI,EX,LAM,CHN,BOI,DS,RL,CAUP,NEK2,TSH,TUTL,TLD,HH,DYSC,E(SPL)MDELTA-HLH,VG,SCA,GL,OPTIX,ATO,DAC |
| GO:0051171 | regulation of nitrogen compound metabolic process | 0.00750148 | 0.00750148 | 1 | SSRP,DG,YETI,EX,TOLLO,CG40191,LAM,DCP2,CHN,INCENP,CG1965,FZY,JUMU,BRAT,EIP63E,ESG,RL,TEFU,MSH6,CAUP,CG12299,CKS30A,L(3)NEO38,CG43366,CG5466,TSH,AB,TET,PDM3,NET,AP,CG13928,CG6654,TIO,EN,HH,SOX15,E(SPL)MGAMMA-HLH,FNE,DLL,NUB,AL,DVE,E(SPL)M8-HLH,NAB,RN,TEY,DOC1,E(SPL)MDELTA-HLH,VG,CG43347,PDM2,SMOG,CG10151,DOC2,SCR,DAN,FD96CA,SP1,GL,LOV,BTD,OPTIX,SCRT,TFAP-2,FD96CB,ATO,SIDPN,DAC,CAS,TUT,TOY,DANR,NERFIN-1,ADA1-1 |
| GO:0034654 | nucleobase-containing compound biosynthetic process | 0.00920398 | 0.00920398 | 1 | LAM,CHN,RPII140,MCM6,CG1965,JUMU,BRAT,EIP63E,RAD50,ESG,RL,TEFU,CAUP,CG12299,RPB12,L(3)NEO38,CG5466,TSH,AB,TET,PDM3,NET,AP,CG6654,TIO,EN,SOX15,E(SPL)MGAMMA-HLH,DLL,NUB,AL,DVE,E(SPL)M8-HLH,NAB,RN,TEY,DOC1,E(SPL)MDELTA-HLH,VG,CG43347,PDM2,CG10151,DOC2,SCR,DAN,FD96CA,SP1,GL,LOV,BTD,OPTIX,SCRT,TFAP-2,FD96CB,ATO,SIDPN,DAC,CAS,TOY,DANR,NERFIN-1,ADA1-1 |
| GO:0060255 | regulation of macromolecule metabolic process | 0.0096421 | 0.0096421 | 1 | FT,SPI,SSRP,DG,YETI,EX,TOLLO,CG40191,TOLL-7,LAM,DCP2,CHN,INCENP,CG1965,FZY,CG9125,JUMU,BRAT,EIP63E,ESG,RL,TEFU,MSH6,CAUP,CG12299,CKS30A,L(3)NEO38,CG43366,CG5466,TSH,AB,TET,PDM3,NET,AP,CG13928,CG6654,TIO,EN,HH,SOX15,E(SPL)MGAMMA-HLH,FNE,DLL,NUB,AL,DVE,E(SPL)M8-HLH,NAB,RN,TEY,DOC1,E(SPL)MDELTA-HLH,VG,CG43347,PDM2,SMOG,CG10151,DOC2,SCR,DAN,FD96CA,SP1,GL,UNC-4,LOV,BTD,OPTIX,SCRT,TFAP-2,FD96CB,ATO,SIDPN,DAC,CAS,TUT,TOY,DANR,NERFIN-1,ADA1-1 |
| GO:0008283 | cell population proliferation | 0.01014524 | 0.01014524 | 1 | FT,SPI,EX,LAM,POLO,BRAT,MUD,ESG,RL,PON,TSH,HH,SOX15,E(SPL)MGAMMA-HLH,NUB,E(SPL)MDELTA-HLH,VG,PDM2,WNT4,BTD,TFAP-2,CAS |
| GO:0048592 | eye morphogenesis | 0.01240118 | 0.01240118 | 1 | FT,SPI,EX,LAM,CHN,BOI,DS,RL,CAUP,NEK2,TSH,TUTL,TLD,HH,DYSC,E(SPL)MDELTA-HLH,VG,SCA,GL,OPTIX,ATO,DAC |
| GO:0090596 | sensory organ morphogenesis | 0.01240118 | 0.01240118 | 1 | FT,SPI,EX,LAM,CHN,BOI,DS,RL,CAUP,NEK2,TSH,TUTL,TLD,HH,DYSC,E(SPL)MDELTA-HLH,VG,SCA,GL,OPTIX,ATO,DAC |
| GO:0080090 | regulation of primary metabolic process | 0.01290147 | 0.01290147 | 1 | SSRP,DG,YETI,EX,TOLLO,CG40191,LAM,DCP2,CHN,INCENP,CG1965,FZY,JUMU,BRAT,EIP63E,ESG,RL,TEFU,MSH6,CAUP,CG12299,CKS30A,L(3)NEO38,CG43366,CG5466,TSH,AB,TET,PDM3,NET,AP,CG13928,CG6654,TIO,EN,HH,SOX15,E(SPL)MGAMMA-HLH,FNE,DLL,NUB,AL,DVE,E(SPL)M8-HLH,NAB,RN,TEY,DOC1,E(SPL)MDELTA-HLH,VG,CG43347,PDM2,CG10151,DOC2,SCR,DAN,FD96CA,SP1,GL,LOV,BTD,OPTIX,SNPF,SCRT,TFAP-2,FD96CB,ATO,SIDPN,DAC,CAS,TUT,TOY,DANR,NERFIN-1,ADA1-1 |
| GO:0007473 | wing disc proximal/distal pattern formation | 0.01601977 | 0.01601977 | 1 | TSH,HH,VG |
| GO:0019438 | aromatic compound biosynthetic process | 0.01695483 | 0.01695483 | 1 | LAM,CHN,RPII140,MCM6,CG1965,JUMU,BRAT,EIP63E,RAD50,ESG,OLF413,RL,TEFU,CAUP,CG12299,RPB12,L(3)NEO38,CG5466,TSH,AB,TET,PDM3,NET,AP,CG6654,TIO,EN,SOX15,E(SPL)MGAMMA-HLH,DLL,NUB,AL,DVE,E(SPL)M8-HLH,NAB,RN,TEY,DOC1,E(SPL)MDELTA-HLH,VG,CG43347,PDM2,CG10151,DOC2,SCR,DAN,FD96CA,SP1,GL,LOV,BTD,OPTIX,SCRT,TFAP-2,FD96CB,ATO,SIDPN,DAC,CAS,TOY,DANR,NERFIN-1,ADA1-1 |
| GO:0031323 | regulation of cellular metabolic process | 0.02033865 | 0.02033865 | 1 | SSRP,DG,YETI,EX,TOLLO,CG40191,TOLL-7,LAM,DCP2,CHN,INCENP,CG1965,FZY,JUMU,BRAT,EIP63E,ESG,RL,TEFU,MSH6,CAUP,CG12299,CKS30A,DCP-1,L(3)NEO38,CG43366,CG5466,TSH,AB,TET,PDM3,NET,AP,CG13928,CG6654,TIO,EN,HH,SOX15,E(SPL)MGAMMA-HLH,FNE,DLL,NUB,AL,DVE,E(SPL)M8-HLH,NAB,RN,TEY,DOC1,E(SPL)MDELTA-HLH,VG,CG43347,PDM2,SMOG,CG10151,DOC2,SCR,DAN,FD96CA,SP1,GL,LOV,BTD,OPTIX,SNPF,SCRT,TFAP-2,FD96CB,ATO,SIDPN,DAC,CAS,TUT,TOY,DANR,NERFIN-1,ADA1-1 |
| GO:1901362 | organic cyclic compound biosynthetic process | 0.02049834 | 0.02049834 | 1 | LAM,CHN,RPII140,LBR,MCM6,CG1965,JUMU,BRAT,EIP63E,RAD50,ESG,OLF413,RL,TEFU,CAUP,CG12299,RPB12,L(3)NEO38,CG5466,TSH,AB,TET,PDM3,NET,AP,CG6654,TIO,EN,SOX15,E(SPL)MGAMMA-HLH,DLL,NUB,AL,DVE,E(SPL)M8-HLH,NAB,RN,TEY,DOC1,E(SPL)MDELTA-HLH,VG,CG43347,PDM2,CG10151,DOC2,SCR,DAN,FD96CA,SP1,GL,LOV,BTD,OPTIX,SCRT,TFAP-2,FD96CB,ATO,SIDPN,DAC,CAS,TOY,DANR,NERFIN-1,ADA1-1 |
| GO:0009791 | post-embryonic development | 0.02212053 | 0.02212053 | 1 | FT,SPI,DG,CHN,RHOGAP54D,JUMU,EIP63E,DS,ESG,RL,CAUP,CKS30A,TYN,OTK,TSH,TOK,NET,AP,EN,TLD,HH,DLL,AL,DVE,RN,VG,LCP9,SCA,SP1,BTD,TFAP-2,ATO,DAC,CAS |
| GO:0050793 | regulation of developmental process | 0.02493125 | 0.02493125 | 1 | FT,SPI,PAV,EX,ROBO1,CHN,PBL,BABOS,POLO,SMC2,BRAT,DS,ESG,RL,TYN,SEMA2B,FUTSCH,TSH,TUTL,HH,SDR,E(SPL)MGAMMA-HLH,NUB,E(SPL)M8-HLH,VMAT,NWK,DYSC,E(SPL)MDELTA-HLH,SMOG,WNT4,BTD,SNPF,SIDPN,CAS,NERFIN-1 |
| GO:0007594 | puparial adhesion | 0.02523827 | 0.02523827 | 1 | SGS7,EIG71EE,SGS3,SGS5 |
| GO:0022608 | multicellular organism adhesion | 0.02523827 | 0.02523827 | 1 | SGS7,EIG71EE,SGS3,SGS5 |
| GO:0022609 | multicellular organism adhesion to substrate | 0.02523827 | 0.02523827 | 1 | SGS7,EIG71EE,SGS3,SGS5 |
| GO:0035215 | genital disc development | 0.02884448 | 0.02884448 | 1 | SPI,ESG,OTK,EN,HH,DLL,TFAP-2,DAC |
| GO:0007548 | sex differentiation | 0.02986675 | 0.02986675 | 1 | ROBO1,LAM,SLI,OTK,EN,HH,DLL,WNT4,ROBO3,TFAP-2,DAC |
| GO:0007479 | leg disc proximal/distal pattern formation | 0.03303884 | 0.03303884 | 1 | RL,TSH,AP,DLL,DAC |
| GO:0007469 | antennal development | 0.03471101 | 0.03471101 | 1 | DLL,AL,DAN,ATO,DAC,DANR |
| GO:0018130 | heterocycle biosynthetic process | 0.0367052 | 0.0367052 | 1 | LAM,CHN,RPII140,MCM6,CG1965,JUMU,BRAT,EIP63E,RAD50,ESG,RL,TEFU,CAUP,CG12299,RPB12,L(3)NEO38,CG5466,TSH,AB,TET,PDM3,NET,AP,CG6654,TIO,EN,SOX15,E(SPL)MGAMMA-HLH,DLL,NUB,AL,DVE,E(SPL)M8-HLH,NAB,RN,TEY,DOC1,E(SPL)MDELTA-HLH,VG,CG43347,PDM2,CG10151,DOC2,SCR,DAN,FD96CA,SP1,GL,LOV,BTD,OPTIX,SCRT,TFAP-2,FD96CB,ATO,SIDPN,DAC,CAS,TOY,DANR,NERFIN-1,ADA1-1 |
| GO:0006928 | movement of cell or subcellular component | 0.03732539 | 0.03732539 | 1 | SPI,DG,PAV,EX,ROBO1,TOLL-7,PBL,KLP61F,STAI,KLP67A,SLI,MUD,ESG,TYN,SEMA2B,FUTSCH,OTK,AB,TEN-A,PDM3,TOK,AP,TRBD,TUTL,EN,CG5142,HH,NRT,DYSC,SMOG,WNT4,ROBO3,CG17083,DSCAM4,DAC,NERFIN-1 |
| GO:0050896 | response to stimulus | 0.03858009 | 0.03858009 | 1 | BARK,FT,SPI,DG,PAV,EX,MCM2,TOLLO,ROBO1,CG9008,TOLL-7,LAM,PBL,RRP1,DNAPOL-ALPHA180,TEP2,MCM6,CG9231,RHOGAP54D,JUMU,EIP63E,SLI,RAD50,MUD,BOI,DS,ESG,RL,TEFU,STI,ROD,MSH6,RHOGEF4,DCP-1,CYP6A13,PIGS,NEK2,SEMA2B,FUTSCH,OTK,CG5466,TSH,AB,TEN-A,PDM3,TOK,AP,TRBD,MRE11,RAB27,TOM,TUTL,EN,TLD,HH,SDR,NRT,HSP67BA,OBP57D,NUB,TOTA,RPK,VMAT,DYSC,NA,CG11438,DOPECR,CCKLR-17D3,COROLLA,GABA-B-R3,E(SPL)M4-BFM,SMOG,BRD,WNT4,NMDAR1,ROBO3,WRY,GLUCLALPHA,SCA,DSCAM4,DOP1R2,OCHO,CSAS,NACHRBETA2,CNGL,NPF,NACHRALPHA1,GYC89DB,GL,LOV,SNPF,GAD1,NACHRBETA1,DAC,NACHRALPHA5,NACHRALPHA6,EIG71EE,NERFIN-1,CG32640,HUG |
| GO:0007476 | imaginal disc-derived wing morphogenesis | 0.03916614 | 0.03916614 | 1 | FT,SPI,DG,JUMU,DS,RL,CAUP,TYN,TSH,TOK,NET,AP,EN,TLD,HH,DLL,DVE,VG,SCA |
| GO:0019222 | regulation of metabolic process | 0.04364332 | 0.04364332 | 1 | FT,SPI,SSRP,DG,YETI,EX,TOLLO,CG40191,TOLL-7,LAM,DCP2,CHN,INCENP,CG1965,FZY,CG9125,JUMU,BRAT,EIP63E,ESG,RL,TEFU,MSH6,CAUP,CG12299,CKS30A,DCP-1,L(3)NEO38,CG43366,CG5466,TSH,AB,TET,PDM3,NET,AP,CG13928,CG6654,TIO,EN,HH,SOX15,E(SPL)MGAMMA-HLH,FNE,DLL,NUB,AL,DVE,E(SPL)M8-HLH,NAB,RN,TEY,DOC1,E(SPL)MDELTA-HLH,VG,CG43347,PDM2,SMOG,CG10151,DOC2,SCR,DAN,FD96CA,SP1,GL,UNC-4,LOV,BTD,OPTIX,SNPF,SCRT,TFAP-2,FD96CB,ATO,SIDPN,DAC,CAS,TUT,TOY,DANR,NERFIN-1,ADA1-1 |
| GO:0000915 | actomyosin contractile ring assembly | 0.04586812 | 0.04586812 | 1 | PAV,LAM,PBL,POLO,STI |
| GO:0001708 | cell fate specification | 0.04769894 | 0.04769894 | 1 | CHN,ESG,TOM,TLD,HH,RN,DOC1,E(SPL)M4-BFM,BRD,DOC2,DAC |
